# Educational technology on chronic non-communicable diseases for Haitian immigrants in the Creole language: a validation study*

**DOI:** 10.1590/1518-8345.8103.4921

**Published:** 2026-10-16

**Authors:** Olbichoo Lexius, Maria de Nazaré de Souza Ribeiro, Cleisiane Xavier Diniz, Elizabeth Teixeira, Fátima Helena do Espírito Santo, Aldalice Aguiar de Souza

**Affiliations:** 1 Universidade do Estado do Amazonas, Escola Superior em Ciências da Saúde, Manaus, AM, Brazil.; 2 Universidade Federal da Bahia, Instituto de Saúde Coletiva, Salvador, BA, Brazil.; 3 Scholarship holder at the Fundação de Amparo à Pesquisa do Estado do Amazonas (FAPEAM), Brazil.; 4 Universidade Federal Fluminense, Escola de Enfermagem Aurora de Afonso Costa, Niterói, RJ, Brazil.

**Keywords:** Noncommunicable Diseases, Emigrants and Immigrants, Validation Study, Educational Technology, Nursing, Health Education

## Abstract

**Objective:**

to validate the content and semantics of an educational technology in the Creole language, in the form of a guide, for the prevention and self-care of Chronic Non-Communicable Diseases for Haitian immigrants.

**Method:**

this is a validation study of educational technology with regard with its content and semantic attributes. There were 17 healthcare professionals experts (Portuguese version) and 30 Haitian immigrants (Creole version) who took part in the content validation. In the content validation, an instrument was used that assessed the target domains, structure/presentation and content relevance of the guide by means of a four-point ordinal scale, and the Concordance Index was sought in the analysis; a semantic validation was performed using the Suitability Assessment of Materials tool, and an analysis was performed using the SAM score.

**Results:**

the overall Concordance Index attribute was 91%; and for the semantic attribute, the SAM score was of 25.8 points.

**Conclusion:**

the educational technology received content and semantic validation, indicating its appropriateness for mediating educational actions of Chronic Non-Communicable Disease prevention and self-care among Haitian immigrants.

## Highlights

**(1)** The Guide has demonstrated technological relevance, validated as adequate.

**(2)** The validated technology is a scientific tool adapted to the immigrant context.

**(3)** The educational technology, in the Creole language, will mediate educational actions for Haitians.

## Introduction

Cultural and linguistic differences, difficulties in dealing with the rules of an unfamiliar health system, with different rules and routines, associated with social vulnerabilities, resulting from undetermined citizenship status, are important factors that hinder us from integration and access to health services from immigrants in Brazil, mainly those who speak a language quite different from Portuguese, as it happens with the Haitians, who have Creole as their mother tongue and French as an additional language.

In Brazil, immigrants have direct access to our health services, therefore, cultural and linguistic diversity compromises the quality of care offered by health services, as elsewhere in the world^1^ ^)-(^ ^3^. Language is one of the major limitations of health professionals not attending to the immigrant and a major barrier to access of these individuals to the health system, making communication difficult for both^4^ ^)-(^ ^6^.

As interactions between migration processes and health are marked by complexity and multiple factors, returning immigrants are particularly vulnerable to difficulties faced. It is fundamental to understand and recognize their particularities, in order to promote improvements in their quality of life, ensure equality in access to health and ensure dignity in care. This includes measures to combat inequalities, discrimination and stigma, as well as the development and improvement of public policies. It is also essential to offer services that meet the current needs of immigrants^7^ ^)-(^ ^10^.

Since 2010, as there has been two more devastating earthquakes in Haiti, the capital of the Amazon has been receiving a large influx of Haitian immigrants. These individuals entered Brazil across the northern borders and, subsequently, migrated to other parts of the country in search of work and income opportunities^9^ ^),(^ ^11^. Among them are those without permanent visa with communication difficulties (they speak Creole and a few speak French), and who form a social group with various significant challenges for the Brazilian Unified Health System^9^ ^),(^ ^12^.

In general, immigrants will become more socially and economically vulnerable by subjecting themselves more easily to unhealthy work, low wages and inhospitable conditions. Such factors contribute to increased risk of malnutrition, occupational diseases and infectious diseases such as tuberculosis, hepatitis, HIV, Chronic Non-Communicable Diseases (NCDs), and psychosocial disorder resulting from social changes and estrangement from family^8^ ^),(^ ^13^ ^)-(^ ^14^.

Regarding Non-Communicable Chronic Diseases (NCDs), it is noteworthy that they are a set of diseases with diverse and modifiable causes and risk factors, with a prolonged latency and course period, often resulting in functional disabilities. Evidence indicates that deaths from NCDs are associated with diseases of the circulatory system (cardiovascular diseases, CVD), chronic respiratory diseases, cancer, and diabetes^15^ ^)-(^ ^16^. In this context, health education initiatives that promote self-care are appropriate.

Health education, as an important strategy for disseminating information and guidance, helps individuals, families, and communities improve their health, enriching their knowledge and affecting their attitude towards well-being and self-care^17^ ^)-(^ ^19^. Self-care is a regulatory human function that people perform on their own or carried out by them based on guidance, to preserve life, health, development, and well-being^20^ ^)-(^ ^21^. Promoting and maintaining self-care among people affected by NCDs is a fundamental role of health professionals, especially nurses, due to their constant interaction with people. In order to mediate the professionals’ actions, educational technologies in health can be used.

Educational technologies in health can promote autonomy, strengthen knowledge building, and thus be feasible for health promotion, risk reduction, and vulnerability prevention^22^ ^)-(^ ^24^. They can also boost health education and provide essential information to alleviate doubts, in order to change risky attitudes by expanding the level of knowledge of the subjects involved^17^ ^),(^ ^25^ ^)-(^ ^27^.

Considering these perspectives, an integrative literature review was conducted to answer the following question: what are the most prevalent non-communicable chronic diseases in the Haitian population? Twenty-five references that addressed the research question were selected from 22 journals. Four NCDs were identified as the most prevalent, namely: hypertension, diabetes, breast cancer and cervical cancer. Based on the results, an educational technology entitled “Guide to prevention and self-care of Non-Communicable Chronic Diseases for Haitian immigrants living in Manaus, Amazonas, Brazil” was developed. The construction was anchored in aspects such as clarity and accessible language, with a view to making the guide attractive and understandable^28^.

For the development of the Guide’s content, it was decided to use official national and international manuals and guidelines on NCDs from, among others, the Brazilian Diabetes Society, the National Cancer Institute (INCA), the Brazilian Association for the Study of Obesity and Metabolic Syndrome (ABESO), the Brazilian Society of Cardiology (SBC), the Ministry of Health, the World Health Organization (WHO), the Pan American Health Organization (PAHO), the Brazilian Society of Mastology (SBM), the U.S. Department of Health and Human Services, the Centers for Disease Control and Prevention (CDC), the American Diabetes Association (ADA).

It is important to point out that an educational technology must be properly developed, accurate, relevant, well understood, and validated before its use^29^ ^)-(^ ^31^. One of the fundamental phases is validation, carried out through expert opinions on the subject (content validation) and the understanding of the text by the target audience (semantic validation)^29^ ^),(^ ^31^ ^)-(^ ^33^. 

Considering the above, the following research question was formulated: is the developed technology, in terms of content (Portuguese version) and semantics (Creole language version), suitable for use? In this sense, the study aimed to validate the content and semantics of an educational technology guide on the prevention and self-care of Non-Communicable Chronic Diseases for Haitian immigrants.

## Method

### Study design

This is a validation study for an educational technology guide in relation to its content attributes (Portuguese version) and semantics (Creole version)^33^. After validating the version in Portuguese, one of the researchers (Haitian) translated it into Creole and five native speakers performed the language review. After the two validations, the two final versions of the Guide were prepared.

### Study settings

Content validation was conducted in a virtual environment. Semantic validation was developed in person at the *Fanm Nwa* Association, an association of Black women, immigrants from Haiti, who participate in the association with their families and other Haitian immigrants. The *Fanm Nwa* Association was chosen for its representativeness, for being the only official association of Haitian immigrants in the state of Amazonas, and for having a registry of immigrants. The association is located in the city of Manaus-AM. 

### Period

The content validation process (Portuguese version) took place from January to February 2023; the revision and translation of the Guide into Creole occurred in March of the same year, followed by semantic validation (Creole version) from April to May; the publication of the two final versions of the Guide was in July 2023.

### Selection criteria

Health professionals with an emphasis on family health, collective health and public health (experts) were selected for content validation. The search occurred through the Lattes Platform. Those selected were those who met at least two of the listed criteria: having at least three years of experience in primary care; having scientific production in the area; having participated in events in the last five years; being a specialist, master or doctor in the area of family health, public health, collective health or related areas (cardiology, nephrology, endocrinology).

For semantic validation, Haitian immigrants (target audience) residing in the city of Manaus-AM and registered with the *Fanm Nwa* Association were selected. Those who were at least 18 years old and who could read in Creole were selected. As exclusion criteria for both groups of participants, those who would be considered ineligible were those who: remained without responding until the 15th day after accepting the invitation; communicated to the project coordination any manifestation of psychological distress during the process; presented difficulties in understanding inherent to the validation processes.

### Sample definition

Pasquali’s guidelines were followed for defining the sample for content validation (experts) and semantic validation (target audience), that is, from 6 to 20^34^.

### Study variables

Regarding the experts, the following variables were considered: age, gender, academic background, professional qualifications, and experience in the field. For the target audience: age, gender, occupation, and level of education. 

### Data collection and used instruments

The experts were invited via electronic contact (email obtained from the contact section of the Lattes Platform) through an invitation letter containing the objectives of the study, the rationale, and the importance of their contribution. Those who responded positively were sent a link to the Google Forms^®^ tool containing: the Informed Consent Form (ICF), a version of the guide in Portuguese, the instrument, and information regarding the completion of the documents and submission of suggestions. After sending the link, a period of fifteen days was given for a response. Experts who did not respond within the stipulated time were disregarded.

The target audience was invited by the research team during face-to-face meetings at the headquarters of the *Fanm Nwa* Association. They were approached orally, at which time they received explanations about the objectives of the study, the rationale, and the importance of their contribution. After expressing acceptance, they received the necessary information for participation and, in printed format, the ICF and a version of the guide in Creole. The completion of the instrument and the indication of suggestions took place after the projected presentation of the guide and its individual reading, carried out in three weekend meetings, specifically for this purpose.

The instrument used for content validation (adapted from Brazilian researchers)^35^ assessed the objective domains, structure/presentation and relevance of the guide’s content using a four-point ordinal scale: totally adequate, adequate, partially adequate and inadequate. The instrument had space for the insertion of suggestions. The adaptation carried out was in the wording of the domain items.

The instrument for semantic evaluation [Suitability Assessment of Materials (SAM) adapted by Galdino^36^] assessed the Guide in five domains (content; language; graphic illustrations; cultural adequacy and motivation) with 13 items containing three scores: 0 (inadequate), 1 (partially inadequate) and 2 (adequate). The instrument had space for the insertion of suggestions.

### Data processing and analysis

The data obtained with the two instruments were entered into a spreadsheet created in Excel with double-checking to control for possible data entry errors. Subsequently, they were transferred to the statistical software IBM^®^ Statistical Package for the Social Sciences, version 24.0. In the analysis of the data obtained with the content validation instrument, the Concordance Index (CI) was sought regarding the representativeness in relation to the content by domain (the sum of the items evaluated as “TA” and “A” divided by the total number of responses in the domain addressed) and by the overall value (obtained from the arithmetic mean of the CI per domain). A minimum parameter for content validation was considered to be a 70% CI^33^. The mean and standard deviation were calculated for continuous variables, and absolute and relative frequencies for the variables. In the analysis of the data obtained with the SAM instrument, the SAM Score^34^ was sought. A score equal to or greater than 10 points out of a total of 26 points^33^ was considered. The suggestions obtained in the two validation stages were organized into a table based on the following actions: include, change, reinforce, review.

### Ethical considerations

All ethical requirements established in Resolutions No. 466/2012 and No. 510/2016, and Circular Letter No. 1, of March 3, 2021, which deals with guidelines for research procedures at any stage in a virtual environment from the National Health Council, were met. The study was evaluated and approved with Opinion number 5,859,857 by the Research Ethics Committee of the Higher School of Health Sciences of the State University of Amazonas, through *Plataforma Brasil* (Certificate of Presentation for Ethical Appreciation - CAAE 66058522.0.0000.5016).

## Results

In the content validation, 17 health professionals participated, including 16 nurses and one physician; all with post-graduation in primary care, public health, collective health, and family health. Regarding academic background, four had a post grad degree, six held master’s degrees, and seven held doctorates; the length of practical experience in their respective areas ranged from four to 39 years.

In the objective domain (which evaluates the purposes, goals, or ends that one wishes to achieve with the technology), the CI was 93%. In the structure and presentation domain (which evaluates the overall organization, structure, presentation strategy, coherence, and formatting), the CI reached 87%. It was found that this domain had the lowest CI, as 15 responses obtained PA (partially adequate). The relevance domain (which evaluates the degree of significance of the technology) reached a CI of 94%. In the overall calculation, domains I, II, and III were summed and divided by the number of domains, resulting in an overall CI of 91%. It was found that all domains achieved a satisfactory CI, which indicates the adequacy of the content (Table 1).

**Table 1:** Distribution of two scores and CI* of the responses obtained by two healthcare professionals in each domain. Manaus, AM, Brazil, 2023

1- Objectives - Refers to purposes, goals or ends that you wish to achieve						
Itens of Domain 1	TA^†^	A^‡^	PA^§^	I^||^	TA^†^+A^‡^	CI*
The information/content is consistent with the daily needs of the target audience for the technology.	12	05	-	-	17	100%
The information/content is important for the quality of life and/or work of the target audience for the technology.	15	02	-	-	17	100%
Invites and/or instigates changes in behavior and attitude	10	05	02	-	15	88%
It can circulate within the scientific community of the field.	09	07	01	-	16	97%
It meets the objectives of institutions that work with the target audience of technology.	09	05	03	-	14	82%
Domain 1 - IC* Total = 93%						
**2- Structure/Presentation - Include general organization, structure, presentation strategy, consistency, and formatting**						
**Itens of Domain 2**	**TA** ^†^	**A** ^‡^	**PA** ^§^	**I** ^||^	**TA** ^†^+A^‡^	CI*
The technology is appropriate for the target audience.	11	05	01	-	16	94%
The messages are presented in a clear and objective manner.	09	07	01	-	16	94%
The information presented is scientifically accurate.	05	10	02	-	15	88%
The material is appropriate to the socio-cultural level of the general public.	08	06	03	-	14	82%
There is a logical sequence of the proposed content	11	05	01	-	16	94%
Information is well structured in agreement and spelling	07	04	06	-	11	64%
The style of the drafting corresponds to the level of knowledge of the audience.	07	09	01	-	16	94%
Domain 2 - IC* Total = 87%						
**3- Relevance - degree of significance of technology**						
**Itens of Domain 3**	**TA** ^†^	**A** ^‡^	**PA** ^§^	**I** ^||^	**TA** ^†^+A^‡^	CI*
These themes portray key-aspects to be strengthened.	13	03	01	-	16	94%
Technology enables generalization and transfer of learning to different contexts.	13	03	01	-	16	94%
Technology propels knowledge building.	13	02	02	-	15	88%
Technology addresses the topics necessary for the target audience to gain knowledge.	12	04	01	-	16	94%
The technology is suitable for use by any professional with the target audience.	08	09	-	-	17	100%
Domain 3 - IC* Total = 94%						
$\text{OVERALL CI}*:\frac{93\%\left(\text{Domain}\;1\right)+87\%\left(\text{Domain}\;2\right)+94\%\left(\text{Domain}\;3\right)}{3}=91\%$						

*CI = Concordance Index; ^†^TA = Totally Adequate; ^‡^A = Adequate; ^§^PA = Partially Adequate; ^||^I = Inadequate

Suggestions obtained from content validation were organized according to steps^37^ to be implemented: include, change, reinforce and review (Figure 1). All suggestions were accepted and considered for the final version (Portuguese).

**Figure 1:** Distribution of the suggestions of two health experts according to the text (Portuguese version). Manaus, AM, Brazil, 2023

Content Attribute	Include:
	• Information from Basic Care Notebook No. 13, page 94, regarding women’s participation in the early detection of breast cancer (HE*- 04)
	• Routine self-examination published by INCA^†^ (Breast awareness) (HE*- 05)
	• Add something graphic/artistic that communicates cultural identification for Haitians (colors of the flag, symbol of the country or other form of visual cultural approximation) (HE*- 07)
	• Provide the addresses of the Basic Health Units in the guide, according to the zone of Manaus, for health follow-up (HE*- 15).
	• On page 20, include an image demonstrating how to measure blood pressure, as was done in the insulin application section (HE*-13)

	Change:
	• In the DM2^‡^ section, I suggest that risk factors be mentioned before signs and symptoms (HE*-08)

	Reinforce:
	• Be more objective and didactic in the instructions, as there is a lot of writing. The more visual the information, the better the understanding (HE*-16)
	• Focus more on Primary Health Care, the gateway to the Unified Health System, where users can ask questions about health technology and self-care (HE*-12)

	Review:
	• Terms such as Systemic Arterial Hypertension and not just Arterial Hypertension, and review of Portuguese grammar. (HE*- 12)

*HE = Health Expert; ^†^INCA = National Cancer Institute; ^‡^DM2 = Type 2 Diabetes Mellitus

In the semantic validation, 30 Haitian immigrants participated, 18 women and 12 men, aged between 25 and 71 years old, with 13 people in the 51 to 71 age range. Among them, 10 had incomplete high school education; only one had completed high school, five people had completed elementary school, 12 had incomplete elementary school, and two had higher education. Regarding work activities, 14 were street vendors, six performed various services (cleaning, beautification, and sewing), two were teachers, two were businesswomen, two were bricklayers, and four did not perform any paid work activity. In relation to the time in Brazil, 15 reported residing in the country for 3 to 5 years, a time considered short; 13 had been residing in the country for between 6 and 10 years; and only two of them had resided in the country for longer, between 11 and 13 years.

In the semantic validation, of those who evaluated it, 25 participants assigned 26 points (maximum score) and five of them assigned 25 points. The overall score was 775 and the average individual score reached 25.8 points (99.2%). The results showed the semantic adequacy of the guide (Table 2).

**Table 2:** Distribution of scores attributed to Haitian immigrant hair according to the responses obtained in each item, second content, language, graphic illustrations, motivation, and cultural appropriateness. Manaus, AM, Brazil, 2023

Itens of each domain	00	01	02	Points earned
	I*	PA^†^	A^‡^	
**Domain 01 - *Tèks* / Content**				
1.1 *Objektif la klè, li pèmèt ou konprann rapid sa ki nan gid la* (The objective is obvious, facilitating ready understanding of the material).	-	-	60	60
1.2 *Tèks yo abòde enfòmasyon ki gen rapò ak kòman pou evite maladi kwonik non transmisib, ak kòman pou w pran swen tèt ou si w ta genyen yo* (This content addresses related information, helps prevent and take care of Chronic Non-Communicable Diseases).	-	-	60	60
1.3 *Gid la rive konplete objektif yo, pou lektè a ka konprann sa ki ekri yo nan yon tan ki rezonab epitou san konfizyon* (The proposal of the material is limited to the objectives, so that the reader can understand in the time allowed and not confused).	-	-	60	60
Total 180 points (100%)				
**Domain 02 - *Langaj* / Language**				
2.1 *Kantite fraz ki nan tèks yo bon pou pèmèt imigran yo konprann* (The reading level is appropriate for the understanding for Haitian immigrants).	-	-	60	60
2.2 *Jan tèks la ekri a pèmèt li fasil pou yo konprann* (The style of writing facilitates the understanding of texts)*.*	-	-	60	60
2.3 *Vokabilè ki itilize yo se mo ke yo konnen e nou mete siyifikasyon* (vocabulary uses common words).	-	-	60	60
Total 180 points (100%)				
**Domain 03 - *Reprezantasyon Grafik* / Graphic Illustrations**				
3.1 *Tit la kapte atansyon epi montre objektif gid la* (The title attracts attention and portrays the purpose of the material).	-	-	60	60
3.2 *Imaj yo montre mesaj yo klè pou lektè a ka konprann pwen prensipal yo pou kont li, san li pa distrè* (The illustrations present basic visual messages so that the reader can grasp the main points without being distracted).	-	04	52	56
Total 116 points (96,7%)				
**Domain 04 - *Motivasyon* / Motivation**				
4.1 *Tèks yo kominike ak lektè a. Li ap pèmèt yo rezoud pwoblèm yo, fè chwa ak / oswa montre kapasite yo* (Interaction of the text with the reader occurs, prompting them to solve problems, make choices, and/or demonstrate skills).	-	01	58	59
4.*2 Modèl konpòtman ou vle yo byen reprezante oswa byen klè* (Desired behavior patterns modeled or well demonstrated).	-	-	60	60
4.3 *Gen enterè pou rann pwòp tèt yo itil, sa vle di, yo motive moun yo pou yo aprann paske yo kwè ke objektif ak konpòtman yo posib* (There is a motivation for self-efficacy, meaning that people are motivated to learn because they believe that the tasks and behaviors are achievable).	-	-	60	60
Total 179 points (99,4%)				
**Domain 05 - *Akò Kiltirèl* / Cultural Adaptation**				
5.1 *Nan lojik kiltirèl, nan lang ak ekperyans imigran yo gid la byen konstwi* (The material is culturally appropriate to the logic, language, and experience of the audience.).	-	-	60	60
5.2 *Li prezante imaj ak mo ki annakò ak kilti yo* (It presents culturally appropriate images and examples).	-	-	60	60
Total 120 points (100%)				
$\text{Overall Score}\;\frac{77\;\text{points}}{30}=25.8\left(99,2\%\right)$				

*I = Inadequate; ^†^PA = Partially Adequate; ^‡^A = Adequate

The suggestions obtained in the semantic validation were also organized according to actions^37^ to be performed: change and reinforce (Figure 2). All suggestions were accepted and considered for the preparation of the final version (creole language).

**Figure 2:** Distribution of immigrants’ suggestions according to actions to be taken in the text (Creole language version). Manaus, AM, Brazil, 2023

Semantic Attribute	Change:
	‘’natirèl’’ oswa ‘’Bio’’ nan plas ‘’Entegral’’ (Put the word “natural” or “Organic” instead of “Integral”) (HI*- 02)

	Reinforce:
	*Mwen sijere ou konstwi pwochèn gid, nenpòt zouti an ak yon nitrisyonis pou w ka mete ki fri espesifik moun ki fè maladi sik oswa tansyon wo pa ka manje paske mwen tande gen fri ki monte sik ak tansyon*
	When you develop the next Guide or any tool, do it together with a nutritionist, in order to include the specific fruits that people with diabetes or hypertension cannot eat because I’ve heard that some fruits increase blood sugar and blood pressure) (HI*- 02)

*HI = Haitian Immigrant

The final version, after the two validations, was structured with the following elements: cover, cataloging data sheet, drafting team, presentation of the Guide, description of the four NCDs (Hypertension, Diabetes, Breast Cancer and Cervical Cancer), final considerations and references. Emphasis was placed on information regarding: prevention of NCDs, signs and symptoms, complications, treatment, recommendations for disease control, access to medication and a link to access the addresses of the nearest health units. In specific sections of the Guide, it was highlighted that the Brazilian Federal Constitution and the SUS guarantee universal access to health for immigrants. Accessible language was used to facilitate comprehension by the target audience; graphic illustrations representative of Haitian culture; and textual and image organization to motivate the use of the Guide, in a creative and proactive way so that there was engagement from the subject^1^ ^-^ ^2^ ^,^ ^25^.

To complement the texts, manually created figures in Figma^®^ by a hired graphic designer were used, in addition to other images obtained from the Canva^®^ collection, ensuring that the content was adapted and visually aligned with the educational and cultural identity purpose (Figure 3). The presence of ethnic-racial representation in health technologies, especially from the Black community, can contribute to improved self-care and increased adherence to treatments^38^ ^-^ ^39^.

**Figure 3:**
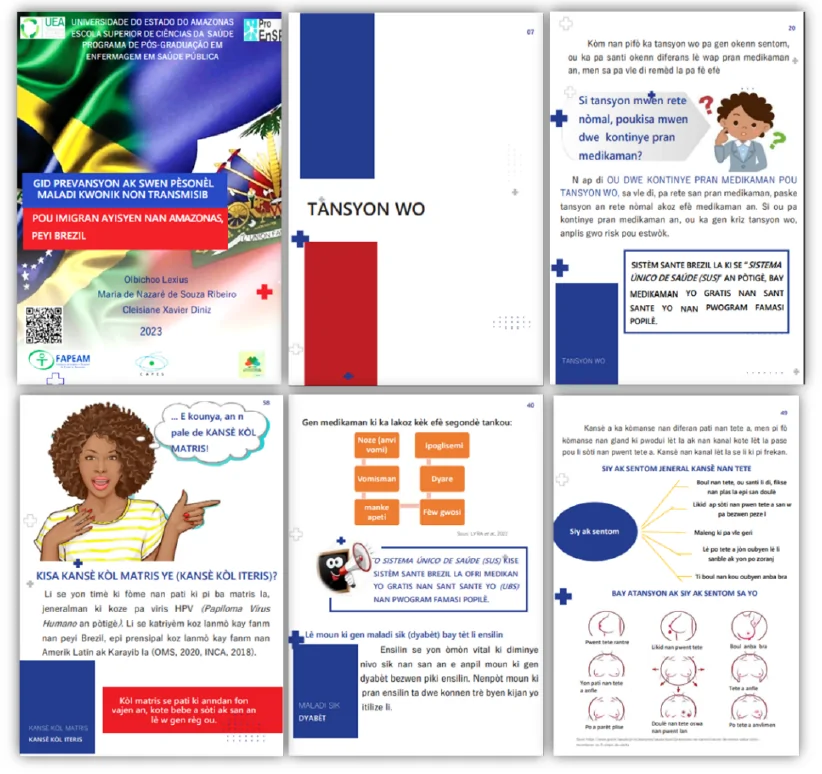
Illustrative image of the Creole Guide entitled “*Gid prevansyon ak swen pèsonèl Maladi Kwonik Non Transmisib pou imigran ayisyen nan Amazonas, peyi Brezil”*

In order to give visibility and provide greater identification, the flags of Brazil and Haiti were placed on the cover; the blue and red colours, present in the Haitian flag, were maintained throughout the opening theme and on the following pages (Figure 3).

The final version of the Guide was completed with 73 pages, due to the font size (14) and the insertion of other images suggested by the evaluators. The final versions in Portuguese and Creole language, in PDF format, are available at: http://educapes.capes.gov.br/handle/capes/735019 e http://educapes.capes.gov.br/handle/capes/734982.

## Discussion

It is undeniable that immigrants experience illness processes with the appearance of NCDs, as a reflection of lifestyles and work in contexts of social vulnerability in receiving countries, which constantly include situations of effective health risks, characteristic of their insertion as immigrants in society^7^.

In 2019, the International Council of Nurses (ICN) reiterated the role of nurses in the “Health for All” program, emphasizing their importance in determining the health needs of their patients and teaching them healthy ways and practices to ensure that they are in optimal health conditions. Thus, nurses and other health professionals are in an excellent position to effect positive changes in the lives of individuals and their families through health education, improving their knowledge, attitudes and behaviors^40^.

The technology now validated constitutes a tool planned and developed based on scientific evidence, in the cultural and social context of Haitian immigrants residing in Brazil, more specifically in the state of Amazonas, and in the health needs of this population. In the applicability of the Guide, the target audience, the scenario and the context were taken into consideration^1^ ^),(^ ^3^ ^)-(^ ^4^ ^),(^ ^6^ ^),(^ ^9^. It is emphasized that all the relevant suggestions were introduced in the second version of the Guide, as indicated in the literature^32^ ^)-(^ ^33^ ^),(^ ^36^.

The overall results of the content validation of the Guide demonstrated agreement and relevance of the technology, evaluating it as adequate, acquiring a score of 91%, higher than the established minimum (70%). Similar studies with diabetes, hypertension, breast cancer and cervical cancer achieved CI of 84%^41^, 84%^42^, 91.71^43^, 95%^44^, respectively.

Regarding semantic validation, the Guide was considered to be of superior quality, with a total score of 99.2%, exceeding the established minimum (70%). Studies that used the same instrument for semantic validation of educational technologies in health obtained very similar values^34^ ^),(^ ^44^ ^)-(^ ^46^. The favorable acceptance of the educational material by Haitian immigrants highlights the importance of personalized strategies, taking into account their individual, ethnic, cultural, and social particularities.

In an analysis of self-management of type 2 Diabetes (DM2), it was found that self-care behaviors are influenced by the individual’s culture, the social support received, and the guidance provided for self-care^47^. In the case of immigrants, it is important to consider health from a holistic perspective, providing support so that they can have the conditions for self-care and access to health services, mainly overcoming the language barrier^7^ ^),(^ ^48^.

In 2021, the Brazilian Ministry of Health launched a Strategic Action Plan to Combat Chronic Non-Communicable Diseases in Brazil 2021-2030, including actions to control cardiovascular diseases, diabetes, cancer, and chronic respiratory disease, by controlling smoking, physical inactivity, inadequate diet, and harmful use of alcohol^49^ ^)-(^ ^50^. Given the growing trend of the Haitian population with NCDs, especially cardiovascular diseases, which has been occurring in recent decades^51^ ^)-(^ ^52^, as well as the vulnerabilities of Haitian immigrants in Manaus, there is a need for strategic innovations to better serve them.

The deficit in the ability to interpret and use health information can affect the maintenance of already established NCDs or their appearance. The implementation of self-care actions promotes collaboration between nurses and individuals/families, so that they develop skills and knowledge to adapt and make informed decisions regarding their chronic disease^53^ ^)-(^ ^54^.

For people living with chronic conditions, managing such conditions involves interacting with information about the disease as well as diagnosis, treatment, lifestyle changes, and better interaction with health professionals and the health system^55^ ^)-(^ ^57^.

Regarding the target audience profile, individuals with lower socioeconomic status were identified, mostly black women. Research indicates that individuals with such characteristics usually have greater difficulty accessing services and receiving health information; and black women are 8% more likely to exhibit low comprehension of information, concluding that low socioeconomic status and skin colour are determining factors for access to and use of health services^57^.

Observing the health literacy of the target audience, with the participation of health specialists and users, the development of educational technologies promotes the co-production of health and can lead to an improvement in the quality, safety, and fairness of care, in addition to favoring collective learning, among other benefits^58^.

This study is subject to some limitations: 1) The sample of the Haitian immigrant group may not be representative of the Haitian population with limited health literacy and reading difficulties; and 2) Only four NCDs were addressed in order to avoid making the content too extensive.

Therefore, it is suggested that future studies evaluate the effectiveness of using this technology in the daily routine of the Haitian population, targeting the four NCDs that were addressed in the Guide, concluding the method steps with the Technology Application phase.

Regarding the relevance of the study for nursing, the Guide, which was constructed and validated and is available in Portuguese and Creole, contributes to improving communication, breaking down stereotypes and prejudices related to this population, and offering more humanized care, aligning with the principles of universality, comprehensiveness, and equity of the Brazilian Unified Health System (SUS). It is worth highlighting that the presence of immigrants of various nationalities and languages in the country requires nurses to have training that makes communication more efficient, ensuring that the immigrant is understood and that information regarding their health is delivered clearly. Since this is not possible, mainly due to linguistic and cultural barriers, other devices adapted to the needs of immigrants should be made available and accessible to them.

In addition, this study contributes significantly to the development of nursing science by producing and validating a Guide on NCDs that brings together updated, varied and reliable information, intended for Haitian immigrants. Its use will make it possible to reduce inadequate self-care and prevention practices.

## Conclusion

The Guide was developed based on national and international guidelines on the most prevalent NCDs in the Haitian population, gathered from an integrative literature review, and improved based on suggestions from participants and scientific sources. It was validated according to content and semantics, the evaluation process of which included 17 health experts and 30 Haitian immigrants residing in Manaus, Amazonas.

In the content validation, an overall CI of 91% was obtained, with all three attributes (objectives, structure/presentation, and relevance) evaluated as satisfactory. In the semantic validation, the overall score reached 25.8 (99.2%) out of a total of 26 points, endorsing the Guide as satisfactorily adequate in terms of content, language, graphic illustrations, motivation, and cultural appropriateness.

The technology, in the Creole language, evaluated as appropriate, can mediate educational actions for the prevention and self-care of Non-Communicable Chronic Diseases for Haitian immigrants. In the final version of the guide, cultural appropriateness was considered, seeking to avoid biases associated with the understanding and conceptualization of health problems, normally experienced by the target audience, aware that cultural and linguistic adaptations are potential barriers to access to health systems and the quality of health care, and impact problems related to the occurrence of NCDs and their complications.

Furthermore, this research contributes to the transfer of knowledge and dissemination of information to populations in vulnerable situations.

## Data Availability

All data generated or analysed during this study are included in this published article.
