# Supplementary material for: Educational technology on chronic non-communicable diseases for Haitian immigrants in the Creole language: a validation study
Source: Rev Lat Am Enfermagem. 2026 Oct 16;34:e4921. doi: 10.1590/1518-8345.8103.4921 (PMC13674969; doi:10.1590/1518-8345.8103.4921)
Supplement: Translation

# Translation: Tecnologia educativa sobre doenças crônicas não transmissíveis para imigrantes haitianos na língua crioula: estudo de validação*

**DOI:** 10.1590/1518-8345.8103.4922

**Authors:** Olbichoo Lexius, Maria de Nazaré de Souza Ribeiro, Cleisiane Xavier Diniz, Elizabeth Teixeira, Fátima Helena do Espírito Santo, Aldalice Aguiar de Souza

**Affiliations:** 1 Universidade do Estado do Amazonas, Escola Superior em Ciências da Saúde, Manaus, AM, Brasil.; 2 Universidade Federal da Bahia, Instituto de Saúde Coletiva, Salvador, BA, Brasil.; 3 Bolsista da Fundação de Amparo à Pesquisa do Estado do Amazonas (FAPEAM), Brasil.; 4 Universidade Federal Fluminense, Escola de Enfermagem Aurora de Afonso Costa, Niterói, RJ, Brasil.

**Keywords:** Doenças Não Transmissíveis, Emigrantes e Imigrantes, Estudo de Validação, Tecnologia Educacional, Enfermagem, Educação em Saúde

## Abstract

**Objetivo:**

validar conteúdo e semântica de uma tecnologia educativa na língua crioula, em formato de guia, para prevenção e autocuidado de Doenças Crônicas Não Transmissíveis para imigrantes haitianos.

**Método:**

trata-se de um estudo de validação de tecnologia educativa no que tange aos atributos conteúdo e semântica. Participaram da validação de conteúdo 17 especialistas da área da saúde (versão português) e da validação semântica 30 imigrantes haitianos (versão em língua crioula). Na validação do conteúdo, utilizou-se um instrumento que avaliou os domínios objetivos, estrutura/apresentação e relevância do conteúdo do guia por meio de uma escala ordinal com quatro pontos, e na análise buscou-se o Índice de Concordância; na validação semântica utilizou-se o instrumento *Suitability Assessment of Materials*, e na análise buscou-se o escore SAM.

**Resultados:**

no atributo conteúdo o Índice de Concordância geral foi 91%; no atributo semântica o escore SAM obtido foi de 25,8 pontos.

**Conclusão:**

a tecnologia educativa obteve validação de conteúdo e semântica, indicando estar apropriada para mediar ações educativas de prevenção e autocuidado de Doenças Crônicas Não Transmissíveis entre imigrantes haitianos.

## Destaques

**(1)** O Guia mostrou-se de relevância tecnológica, avaliada como adequada.

**(2)** A tecnologia validada é uma ferramenta científica adaptada ao contexto dos imigrantes.

**(3)** A tecnologia, na língua crioula, poderá mediar ações educativas para haitianos.

## Introdução

As diferenças culturais e linguísticas, as dificuldades em lidar com as normas de um sistema de saúde desconhecido, com diferentes regras e rotinas, associadas às vulnerabilidades sociais, decorrentes do status indeterminado de cidadão, são importantes fatores que dificultam a integração e o acesso aos serviços de saúde por imigrantes no Brasil, principalmente aqueles que se comunicam em uma língua bastante distinta do português, como ocorre com os haitianos, que têm o crioulo como língua materna e o francês como língua adicional. 

No Brasil, os imigrantes têm direito ao acesso nos serviços de saúde, porém, a diversidade cultural e linguística compromete a qualidade do cuidado oferecido pelos serviços de saúde, como em qualquer outro lugar do mundo^1^ ^)-(^ ^3^. O idioma é uma das maiores limitações dos profissionais de saúde no atendimento ao imigrante e uma importante barreira de acesso desses indivíduos ao sistema de saúde, tornando a comunicação inviável para ambos^4^ ^)-(^ ^6^.

As interações entre os processos migratórios e a saúde são marcadas por uma complexidade e por múltiplos fatores, que tornam os imigrantes particularmente vulneráveis às dificuldades enfrentadas. É fundamental compreender e reconhecer suas particularidades, além de promover melhorias em sua qualidade de vida, garantir igualdade no acesso à saúde e assegurar a dignidade no atendimento. Isso inclui ações para combater desigualdades, discriminações e estigmas, assim como o desenvolvimento e a melhoria das políticas públicas. Também é essencial oferecer serviços que atendam às necessidades atuais dos imigrantes^7^ ^)-(^ ^10^. 

Desde o ano de 2010, em que um dos mais devastadores terremotos atingiu o Haiti, a capital do Amazonas passou a receber um grande fluxo de imigrantes haitianos. Esses indivíduos entraram no Brasil pelas fronteiras do norte e, posteriormente, migraram para outras partes do país em busca de oportunidades de trabalho e renda^9^ ^),(^ ^11^. Dentre eles estão aqueles que não possuem visto permanente, com dificuldade de comunicação (falam o idioma crioulo e poucos falam francês), e que compõem um grupo social com diversos desafios significativos para o Sistema Único de Saúde (SUS)^9^ ^),(^ ^12^.

Os imigrantes, em geral, são mais vulneráveis social e economicamente por submeterem-se mais facilmente a trabalho insalubres, salários baixos e moradias inóspitas. Tais fatores propiciam maior risco de subnutrição, doenças laborais e doenças infecciosas como tuberculose, hepatites, HIV, Doenças Crônicas Não Transmissíveis (DCNT), além de transtornos psicossociais decorrentes das mudanças sociais e do afastamento da família^8^ ^),(^ ^13^ ^)-(^ ^14^.

Em relação às Doenças Crônicas Não Transmissíveis (DCNT), destaca-se que são um conjunto de doenças de causas e fatores de risco diversificados e modificáveis, com período prolongado de latência e de curso, muitas vezes resultando em incapacidades funcionais. As evidências apontam que os óbitos por DCNT são imputados às doenças do aparelho circulatório (DAC), às doenças cônicas do sistema respiratório, ao câncer e ao diabetes^15^ ^)-(^ ^16^. Nesse âmbito, cabem iniciativas de educação em saúde a favor do autocuidado.

A educação em saúde, enquanto importante estratégia de disseminação de informações e orientações, ajuda o indivíduo, família e comunidade a melhorar sua saúde, enriquecendo seus conhecimentos e afetando sua atitude em relação ao bem-estar e autocuidado^17^ ^)-(^ ^19^. O autocuidado é uma função humana reguladora que as pessoas desempenham por si próprias ou executada por elas a partir de orientação, para preservar a vida, a saúde, o desenvolvimento e o bem-estar^20^ ^)-(^ ^21^. Promover e manter o autocuidado com pessoas acometidas de DCNT é papel fundamental da atuação dos profissionais de saúde, principalmente enfermeiros, por sua interação constante com o indivíduo. Para mediar a atuação dos profissionais, pode-se fazer uso de tecnologias educativas em saúde. 

As tecnologias educativas em saúde poderão favorecer a autonomia, fortalecer a construção do conhecimento e, assim, configurar-se como factíveis para a promoção da saúde, redução de riscos e prevenção de vulnerabilidades^22^ ^)-(^ ^24^. Também podem impulsionar a educação em saúde e fornecer informações essenciais para minorar dúvidas, a fim de mudar atitudes de risco ao expandir o nível de conhecimento dos sujeitos envolvidos^17^ ^),(^ ^25^ ^)-(^ ^27^.

Considerando tais perspectivas, realizou-se uma revisão da literatura do tipo integrativa que buscou resposta para a seguinte questão: quais as doenças crônicas não transmissíveis mais prevalentes na população haitiana? Foram selecionadas 25 referências que atenderam à questão da pesquisa, provenientes de 22 periódicos. Identificou-se quatro DCNT (as mais prevalentes): hipertensão, diabetes, câncer de mama e câncer do colo de útero. Com base nos resultados, elaborou-se uma tecnologia educativa intitulada “Guia de prevenção e autocuidado de Doenças Crônicas Não Transmissíveis para imigrantes haitianos, residentes em Manaus, Amazonas, Brasil”. A construção foi ancorada em aspectos como clareza e linguagem acessível, com vistas a tornar o guia atrativo e compreensível^28^.

Para a elaboração do conteúdo do Guia, optou-se por utilizar manuais e diretrizes oficiais nacionais e internacionais sobre DCNT da Sociedade Brasileira de Diabetes, do Instituto Nacional de Câncer (INCA), da Associação Brasileira para o Estudo da Obesidade e da Síndrome Metabólica (ABESO), da Sociedade Brasileira de Cardiologia (SBC), do Ministério da Saúde, da Organização Mundial da Saúde (OMS), da Organização Pan-Americana da Saúde (OPAS), da Sociedade Brasileira de Mastologia (SBM), do *U.S. Department of Health and Human Services*, do *Centers For Disease Controle And Prevention* (CDC), *American Diabetes Association* (ADA), dentre outros.

Uma tecnologia educativa deve ser adequadamente elaborada, precisa, relevante, bem entendida e validada antes de sua utilização^29^ ^)-(^ ^31^. Uma das fases fundamentais é a validação, feita por meio de opiniões de especialistas na temática (validação de conteúdo) e a compreensão do texto pelo público-alvo (validação semântica)^29^ ^),(^ ^31^ ^)-(^ ^33^.

Considerando o exposto, formulou-se a seguinte pergunta de pesquisa: a tecnologia elaborada, no que tange ao conteúdo (versão em português) e à semântica (versão em língua crioula), está adequada para ser utilizada? Nesse sentido, o estudo teve como objetivo validar conteúdo e semântica de uma tecnologia educativa sobre prevenção e autocuidado de Doenças Crônicas Não Transmissíveis para imigrantes haitianos.

## Método

### Tipo de estudo

Trata-se de um estudo de validação de tecnologia educativa segundo os atributos conteúdo (versão português) e semântica (versão em língua crioula)^33^. Após a validação da versão em português, uma das pesquisadoras (haitiana) fez a tradução para a língua crioula e cinco nativos na língua realizaram a revisão linguística. Após as duas validações, procedeu-se à confecção das duas versões finais do Guia.

### Cenários do estudo

A validação de conteúdo foi realizada em ambiente virtual. A validação semântica foi desenvolvida de modo presencial no espaço da Associação *Fanm Nwa*, uma associação de mulheres pretas, imigrantes do Haiti, que participam da associação com seus familiares e outros imigrantes haitianos. A escolha da Associação *Fanm Nwa* se deu por sua representatividade, por ser a única associação oficial de imigrantes haitianos no estado do Amazonas, e por ter um cadastro dos imigrantes. A associação localiza-se na cidade de Manaus-AM. 

### Período

O processo de validação de conteúdo (versão português) ocorreu de janeiro a fevereiro de 2023; a revisão e tradução do Guia para a língua crioula ocorreu em março do mesmo ano, seguindo-se a validação semântica (versão língua crioula) de abril a maio; a publicização das duas versões finais do Guia foi em julho de 2023. 

### Critérios de seleção

Para a validação de conteúdo foram selecionados profissionais da área da saúde com ênfase em saúde da família, saúde coletiva e saúde pública (especialistas). A busca ocorreu por meio da Plataforma Lattes. Foram selecionados aqueles que atenderam a pelo menos dois dos critérios listados: ter no mínimo três anos de experiência na atenção básica; ter produção científica na área; ter participado de eventos nos últimos cinco anos; ser especialista, mestre ou doutor na área de saúde da família, saúde pública, saúde coletiva ou áreas afins (cardiologia, nefrologia, endocrinologia).

Para a validação semântica foram selecionados imigrantes haitianos (público-alvo) residentes na cidade de Manaus-AM e cadastrados na Associação *Fanm Nwa.* Foram selecionados aqueles com no mínimo 18 anos de idade e que soubessem ler em língua crioulo. Como critérios de exclusão para os dois grupos de participantes, seriam considerados inelegíveis aqueles que: permanecessem sem dar resposta até o 15º dia após aceitação do convite; comunicassem à coordenação do projeto manifestação de sofrimento psíquico durante o processo; apresentassem dificuldades de compreensão inerentes aos processos de validação.

### Definição da amostra

Para a definição da amostra para a validação de conteúdo (especialistas) e semântica (público-alvo) foi seguido o que indica Pasquali, ou seja, de 6 a 20^34^.

### Variáveis do estudo

Em relação aos especialistas, as seguintes variáveis foram consideradas: idade, gênero, formação acadêmica, qualificação profissional e experiência na área. Para o público-alvo: idade, gênero, ocupação, grau de instrução. 

### Coleta de dados e instrumentos utilizados

Os especialistas foram convidados mediante contato eletrônico (e-mail obtido na sessão contato da Plataforma Lattes) por meio de uma carta convite contendo os objetivos do estudo, a justificativa e a importância de sua contribuição. Aos que responderam positivamente, foi enviado link da ferramenta Google *Forms* ^*®*^ contendo: Termo de Consentimento Livre e Esclarecido (TCLE), uma versão do guia em português, instrumento e informações quanto ao preenchimento dos documentos e encaminhamento de sugestões. Após envio do *link*, foi dado prazo de quinze dias para retorno. Os especialistas que não responderam no tempo estipulado foram desconsiderados. 

O público-alvo foi convidado pela equipe de pesquisa durante reuniões presenciais na sede da Associação *Fanm Nwa*. Foram abordados oralmente, momento em que receberam explicações sobre os objetivos do estudo, a justificativa e a importância da sua contribuição. Após manifestarem aceitação, receberam as informações necessárias para a participação e em formato impresso o TCLE e uma versão do guia em língua crioula. O preenchimento do instrumento e a indicação de sugestões se deu após a apresentação projetada do guia e sua leitura individual, realizado em três encontros em finais de semana, especificamente para este fim.

O instrumento utilizado para validação de conteúdo (adaptado de pesquisadores brasileiros)^35^ ^)^ avaliou os domínios objetivos, estrutura/apresentação e relevância do conteúdo do guia por meio de uma escala ordinal com quatro pontos: totalmente adequado, adequado, parcialmente adequado e inadequado. O instrumento tinha espaço para inserção de sugestões. A adaptação realizada foi na redação dos itens dos domínios.

O instrumento para avaliação semântica [*Suitability Assessment of Materials* (SAM) adaptado por Galdino^36^] avaliou o guia em cinco domínios (conteúdo; linguagem; ilustrações gráficas; adequação cultural e motivação) com 13 itens contendo três pontuações: 0 (inadequado), 1 (parcialmente inadequado) e 2 (adequado). No instrumento havia espaço para inserção de sugestões.

### Tratamento e análise dos dados

Os dados obtidos com os dois instrumentos foram lançados em uma planilha construída no Excel com dupla conferência para controlar possíveis erros de alimentação do banco de dados. Posteriormente, foram transportados para o *software* estatístico IBM^®^ *Statistical Package for the Social Sciences* versão 24.0. Na análise dos dados obtidos com o instrumento de validação de conteúdo, buscou-se o Índice de Concordância (IC) quanto à representatividade em relação ao conteúdo por domínio (somou-se os itens avaliados como “TA” e “A” divididos pelo número total de respostas do domínio abordado) e pelo valor global (obtido da média aritmética do IC por domínio). Considerou-se como parâmetro mínimo de validação de conteúdo atingir-se 70% de IC^33^. A média e o desvio-padrão foram calculados para variáveis contínuas, frequência absoluta e relativa para as variáveis. Na análise dos dados obtidos com o instrumento SAM, buscou-se o Escore SAM^34^. Considerou-se um escore igual ou superior a 10 pontos do total de 26 pontos^33^. As sugestões obtidas nas duas etapas de validação foram organizadas em um quadro a partir da menção às seguintes ações: incluir, alterar, reforçar, revisar. 

### Considerações éticas

Todos os requisitos éticos estabelecidos nas Resoluções Nº 466/2012 e Nº 510/2016 e Carta Circular nº 1, de 3 de março de 2021 que trata de orientações para procedimentos em pesquisas com qualquer etapa em ambiente virtual do Conselho Nacional de Saúde, foram atendidos. O estudo foi avaliado e aprovado com o Parecer 5.859.857, pelo Comitê de Ética em Pesquisa da Escola Superior de Ciências da Saúde da Universidade do Estado do Amazonas, via Plataforma Brasil (CAAE 66058522.0.0000.5016).

## Resultados

Na validação de conteúdo participaram 17 profissionais, sendo 16 enfermeiras(os) e um médico; todos com especialização em atenção primária, saúde pública, saúde coletiva e da família. Quanto à formação acadêmica, quatro especialistas, seis mestres e sete doutores; o tempo de experiência prática nas respectivas áreas foi de quatro a 39 anos. 

No domínio objetivo (que avalia propósitos, metas ou fins que se deseja atingir com a tecnologia), o IC foi de 93%. No domínio estrutura e apresentação (que avalia a organização geral, estrutura, estratégia de apresentação, coerência e formatação) o IC atingiu 87%. Constatou-se que este domínio apresentou o menor IC, pois 15 respostas obtiveram PA (parcialmente adequado). O domínio relevância (que avalia o grau de significação da tecnologia) alcançou IC 94%. No cálculo geral foram somados os domínios I, II e III divididos pelo número de domínios, obtendo-se IC geral 91%. Constatou-se que todos os domínios alcançaram IC satisfatório, o que indica adequação do conteúdo (Tabela 1).

**Tabela 1:** Distribuição dos escores e IC* das respostas obtidas dos especialistas da área da saúde em cada domínio. Manaus, AM, Brasil, 2023

1-Objetivos - Referem-se a propósitos, metas ou fins que se deseja atingir						
Itens do Domínio 1	TA^†^	A^‡^	PA^§^	I^||^	TA^†^+A^‡^	IC*
As informações/conteúdos são ou estão coerentes com as necessidades cotidianas do público-alvo da tecnologia.	12	05	-	-	17	100%
As informações/conteúdos são importantes para a qualidade de vida e/ou o trabalho do público-alvo da tecnologia.	15	02	-	-	17	100%
Convida e/ou instiga a mudanças de comportamento e atitude	10	05	02	-	15	88%
Pode circular no meio científico da área.	09	07	01	-	16	97%
Atende aos objetivos de instituições que trabalham com o público-alvo da tecnologia.	09	05	03	-	14	82%
Domínio 1 - Total do IC* = 93%						
**2-Estrutura/Apresentação - Inclui organização geral, estrutura, estratégia de apresentação, coerência e formatação.**						
**Itens do Domínio 2**	**TA** ^†^	**A** ^‡^	**PA** ^§^	**I** ^||^	**TA** ^†^+A^‡^	IC*
A tecnologia é apropriada para o público-alvo.	11	05	01	-	16	94%
As mensagens estão apresentadas de maneira clara e objetiva.	09	07	01	-	16	94%
As informações apresentadas estão cientificamente corretas.	05	10	02	-	15	88%
O material está apropriado ao nível sociocultural do público-alvo.	08	06	03	-	14	82%
Há uma sequência lógica do conteúdo proposto	11	05	01	-	16	94%
As informações estão bem estruturadas em concordância e ortografia	07	04	06	-	11	64%
O estilo da redação corresponde ao nível de conhecimento do público-alvo.	07	09	01	-	16	94%
Domínio 2 - Total do IC* = 87%						
**3-Relevância- grau de significação da tecnologia**						
**Itens do Domínio 3**	**TA** ^†^	**A** ^‡^	**PA** ^§^	**I** ^||^	**TA** ^†^+A^‡^	IC*
Os temas retratam aspectos-chave que devem ser reforçados.	13	03	01	-	16	94%
A tecnologia permite generalização e transferência do aprendizado a diferentes contextos.	13	03	01	-	16	94%
A tecnologia propõe a construção de conhecimentos.	13	02	02	-	15	88%
A tecnologia aborda os assuntos necessários para o saber do público-alvo.	12	04	01	-	16	94%
A tecnologia está adequada para ser usada por qualquer profissional com o público-alvo.	08	09	-	-	17	100%
Domínio 3 - Total do IC* = 94%						
$IC*\;\text{GERAL}:\frac{93\%\left(\text{Domínio}\;1\right)+87\%\left(\text{Domínio}\;2\right)+94\%\left(\text{Domínio}\;3\right)}{3}=91\%$						

*IC = Índice de Concordância; ^†^TA = Totalmente Adequado; ^‡^A = Adequado; ^§^PA = Parcialmente Adequado; ^||^I = Inadequado

As sugestões obtidas na validação de conteúdo foram organizadas segundo ações^37^ a serem executadas: incluir, alterar, reforçar e revisar (Figura 1). Todas as sugestões foram acatadas e consideradas para a confecção da versão final (português).

**Figura 1:** Distribuição das sugestões dos especialistas da área da saúde segundo ações a realizar no texto (versão português). Manaus, AM, Brasil, 2023

Atributo Conteúdo	Incluir:
	Informações do Caderno de Atenção Básica nº13, página 94, com relação a participação da mulher na detecção precoce do câncer de mama (ES*- 04)
	O autoexame de rotina publicado pelo INCA^†^ (*Breast awareness*) (ES*- 05)
	Acrescentar algo gráfico/artístico que comunique identificação cultural para o haitiano (cores da bandeira, símbolo do país ou outra forma de aproximação visual cultural) (ES*- 07)
	Disponibilizar no guia os endereços das Unidades Básicas de Saúde, conforme zona de Manaus, para acompanhamento de saúde (ES*- 15).
	Na página 20 colocar uma imagem demonstrando como aferir pressão, assim como foi feito na aplicação de insulina (ES*-13)

	Alterar:
	No tema DM2^‡^, sugiro que os fatores de risco sejam citados antes dos sinais e sintomas (ES*-08)

	Reforçar:
	Ser mais objetiva e didática nas orientações, pois tem muita escrita. Quanto mais visual a informação melhor será o entendimento (ES*-16)
	Abordar mais a Atenção Primária à Saúde, porta de acesso do Sistema Único de Saúde, onde o usuário poderá tirar dúvidas sobre a tecnologia de saúde e seu autocuidado (ES*-12)

	Revisar:
	Termos como Hipertensão Arterial Sistêmica e não somente Hipertensão Arterial e revisão de português (ES*- 12)

*ES = Especialista da Saúde; ^†^INCA = Instituto Nacional do Câncer; ^‡^DM2 = Diabetes Mellitus Tipo 2

Na validação semântica participaram 30 imigrantes haitianos, 18 mulheres e 12 homens, na faixa etária entre 25 e 71 anos de idade, sendo 13 pessoas na faixa de 51 a 71 anos. Dentre eles, 10 possuíam o correspondente ao ensino médio incompleto; somente um, ensino médio completo, cinco pessoas possuíam ensino fundamental completo, 12 possuíam ensino fundamental incompleto e dois o ensino superior. No que se refere às atividades laborais, 14 eram vendedores ambulantes, seis realizavam serviços diversos (faxina, embelezamento e costura), duas professoras, duas empresárias, dois pedreiros e quatro não executavam nenhuma atividade laboral remunerada. Em relação ao tempo no Brasil, 15 informaram estar residindo no país por 3 a 5 anos, tempo considerado pequeno; 13 estavam residindo entre 6 a 10 anos; e somente dois deles residiam há mais tempo no país, entre 11 a 13 anos.

Na validação semântica, dos que avaliaram, 25 participantes atribuíram 26 pontos (pontuação máxima) e cinco deles atribuíram 25 pontos. A pontuação geral foi 775 e a média da pontuação individual alcançou 25,8 pontos (99,2%). Constatou-se, a partir dos resultados, a adequação semântica do guia (Tabela 2).

**Tabela 2:** Distribuição das pontuações atribuídas pelos imigrantes haitianos de acordo com as respostas obtidas em cada item, segundo conteúdo, linguagem, ilustrações gráficas, motivação e adequação cultural. Manaus, AM, Brasil, 2023

Itens do domínio	00	01	02	Pontos obtidos
	I*	PA^†^	A^‡^	
**Domínio 01 - *Tèks* / Conteúdo**				
1.1 *Objektif la klè, li pèmèt ou konprann rapid sa ki nan gid la* (O objetivo é evidente, facilitando a pronta compreensão do material).	-	-	60	60
1.2 *Tèks yo abòde enfòmasyon ki gen rapò ak kòman pou evite maladi kwonik non transmisib, ak kòman pou w pran swen tèt ou si w ta genyen yo* (O conteúdo aborda informações relacionadas, ajudem a prevenir e autocuidar das Doenças Crônicas Não Transmissíveis).	-	-	60	60
1.3 *Gid la rive konplete objektif yo, pou lektè a ka konprann sa ki ekri yo nan yon tan ki rezonab epitou san konfizyon* (A proposta do material é limitada aos objetivos, para que o leitor possa compreender no tempo permitido e sem confusão).	-	-	60	60
Total 180 pontos (100%)				
**Domínio 02 - *Langaj* / Linguagem**				
2.1 *Kantite fraz ki nan tèks yo bon pou pèmèt imigran yo konprann* (O nível de leitura é adequado para a compreensão do imigrante haitiano).	-	-	60	60
2.2 *Jan tèks la ekri a pèmèt li fasil pou yo konprann* (O estilo da escrita facilita o entendimento dos textos)*.*	-	-	60	60
2.3 *Vokabilè ki itilize yo se mo ke yo konnen e nou mete siyifikasyon* (O vocabulário utiliza palavras comuns).	-	-	60	60
Total 180 pontos (100%)				
**Domínio 03 - *Reprezantasyon Grafik* / Ilustrações Gráficas**				
3.1 *Tit la kapte atansyon epi montre objektif gid la* (O título atrai a atenção e retrata o propósito do material).	-	-	60	60
3.2 *Imaj yo montre mesaj yo klè pou lektè a ka konprann pwen prensipal yo pou kont li, san li pa distrè* (As ilustrações apresentam mensagens visuais fundamentais para que o leitor possa compreender os pontos principais sozinho, sem distrações).	-	04	52	56
Total 116 pontos (96,7%)				
**Domínio 04 - *Motivasyon* / Motivação**				
4.1 *Tèks yo kominike ak lektè a. Li ap pèmèt yo rezoud pwoblèm yo, fè chwa ak / oswa montre kapasite yo* (Ocorre interação do texto com o leitor, levando-os a resolver problemas, fazer escolhas e/ou demonstrar habilidades).	-	01	58	59
4.*2 Modèl konpòtman ou vle yo byen reprezante oswa byen klè* (Os padrões de comportamento desejados são modelados ou bem demonstrados).	-	-	60	60
4.3 *Gen enterè pou rann pwòp tèt yo itil, sa vle di, yo motive moun yo pou yo aprann paske yo kwè ke objektif ak konpòtman yo posib* (Existe a motivação à autoeficácia, ou seja, as pessoas são motivadas a aprender por acreditarem que as tarefas e comportamentos são factíveis).	-	-	60	60
Total 179 pontos (99,4%)				
**Domínio 05 - *Akò Kiltirèl* / Adequação Cultural**				
5.1 *Nan lojik kiltirèl, nan lang ak ekperyans imigran yo gid la byen konstwi* (O material é culturalmente adequado à lógica, linguagem e experiência do público-alvo).	-	-	60	60
5.2 *Li prezante imaj ak mo ki annakò ak kilti yo* (Apresenta imagens e exemplos adequados culturalmente).	-	-	60	60
Total 120 pontos (100%)				
$\text{Pontuação Geral}\;\frac{775\;\text{pontos}}{30}=25,8\left(99,2\%\right)$				

*I = Inadequado; ^†^PA = Parcialmente Adequado; ^‡^A = Adequado

As sugestões obtidas na validação semântica também foram organizadas segundo ações^37^ a serem executadas: alterar e reforçar (Figura 2). Todas as sugestões foram acatadas e consideradas para a confecção da versão final (língua crioula).

**Figura 2:** Distribuição das sugestões dos imigrantes segundo ações a realizar no texto (versão língua crioula). Manaus, AM, Brasil, 2023

Atributo Semântica	Alterar:
	Mete mo ‘’natirèl’’ oswa ‘’Bio’’ nan plas ‘’Entegral’’(Colocar a palavra ‘’natural’’ ou ‘’Bio’’ no lugar ‘’Integral’’) (IH*- 02)

	Reforçar:
	*Mwen sijere ou konstwi pwochèn gid, nenpòt zouti an ak yon nitrisyonis pou w ka mete ki fri espesifik moun ki fè maladi sik oswa tansyon wo pa ka manje paske mwen tande gen fri ki monte sik ak tansyon.* (Quando você desenvolver o próximo Guia ou qualquer ferramenta, fazê-lo junto com um nutricionista, afim de colocar as frutas específicas que as pessoas diabéticas ou hipertensas não podem comer porque ouvi falar que têm frutas que aumentam a glicemia e a pressão) (IH*- 02)

*IH = Imigrante Haitiano

A versão final, após as duas validações, foi estruturada com os seguintes elementos: capa, ficha catalográfica, equipe de elaboração, apresentação do Guia, descrição das quatro DCNT (Hipertensão Arterial, Diabetes, Câncer de Mama e Câncer do Colo do Útero/Câncer Cervical), considerações finais e referências. Foram enfatizadas as informações referentes a: prevenção das DCNT, sinais e sintomas, complicações, tratamento, recomendações para o controle da doença, acesso à medicação e um link para acessar os endereços de unidades de saúde mais próximas. Em espaços específicos do Guia, foi salientado que a Constituição Federal Brasileira e o SUS garantem aos imigrantes acesso universal à saúde. Utilizou-se uma linguagem acessível para facilitar a compreensão pelo público-alvo; ilustrações gráficas representativas da cultura haitiana; e organização textual e imagética para motivar o uso do Guia, de forma criativa e proativa para que houvesse engajamento do sujeito^1^ ^)-(^ ^2^ ^),(^ ^25^.

Para complementar os textos, foram utilizadas figuras confeccionadas de forma manual no Figma^®^, por uma designer gráfica contratada, além de outras imagens obtidas do acervo do Canva^®^, assegurando que o conteúdo fosse adaptado e visualmente alinhado com o propósito educacional e cultural identitário (Figura 3). A presença de representatividade étnico-racial nas tecnologias de saúde, especialmente da comunidade negra, pode contribuir para um aprimoramento do autocuidado e aumentar a adesão aos tratamentos^38^ ^)-(^ ^39^.

**Figura 3:** Imagem ilustrativa do Guia em língua crioula intitulado “*Gid prevansyon ak swen pèsonèl Maladi Kwonik Non Transmisib pou imigran ayisyen nan Amazonas, peyi Brezil”*

A fim de dar visibilidade e provocar maior identificação, na capa foram colocadas as bandeiras do Brasil e do Haiti; as cores azul e vermelho, presentes na bandeira do Haiti, foram mantidas em toda abertura de tema e nas páginas que se seguem (Figura 3).

A última versão do Guia foi finalizada com 73 páginas, devido ao tamanho da fonte (14) e à inserção de outras imagens sugeridas pelos avaliadores. As versões finais em português e língua crioula, no formato PDF, estão disponíveis em: http://educapes.capes.gov.br/handle/capes/735019 e http://educapes.capes.gov.br/handle/capes/734982.

## Discussão

É incontestável que os imigrantes vivenciam processos de adoecimento com o aparecimento das DCNT, como reflexo dos modos de vida e trabalho em contextos de vulnerabilidade social nos países receptores que, com constância, compreendem situações de risco efetivos à saúde, característicos da sua inserção como imigrantes na sociedade^7^. 

Em 2019, o *International Council of Nurses* (ICN), reiterou o papel dos enfermeiros no programa “Saúde para Todos”, enfatizando sua importância na determinação das necessidades de saúde de seus pacientes e ensinando-lhes maneiras e práticas saudáveis para garantir que eles estejam em ótimas condições saúde Assim, os enfermeiros e outros profissionais de saúde se encontram numa excelente posição para efetuar mudanças positivas na vida dos indivíduos e das suas famílias através da educação em saúde, na melhoria dos seus conhecimentos, atitudes e comportamentos^40^.

A tecnologia ora validada constitui-se como uma ferramenta planejada e desenvolvida com base em evidências científicas, no contexto cultural e social dos imigrantes haitianos que residem no Brasil, mais especificamente no estado do Amazonas, e nas necessidades em saúde deste público. Na aplicabilidade do Guia, levou-se em consideração o público-alvo, o cenário e o contexto^1^ ^),(^ ^3^ ^)-(^ ^4^ ^),(^ ^6^ ^),(^ ^9^. Enfatiza-se que todas as sugestões procedentes foram introduzidas na segunda versão do Guia, como indicado na literatura^32^ ^)-(^ ^33^ ^),(^ ^36^.

Os resultados gerais da validação de conteúdo do Guia demostraram uma concordância e relevância da tecnologia, avaliando-a como adequada, adquirindo um escore de 91%, superior ao mínimo estabelecido (70%). Estudos semelhantes com diabetes, hipertensão, câncer de mama e câncer cervical alcançaram IC de 84%^41^, 84%^42^, 91,71^43^, 95%^44^, respectivamente. 

Em relação à validação semântica, o Guia foi considerado de qualidade superior, com escore total de 99,2%, ficando acima do mínimo estabelecido (70%). Estudos que utilizaram o mesmo instrumento para validação semântica de tecnologias educacionais em saúde obtiveram valores muito próximos^34^ ^),(^ ^44^ ^)-(^ ^46^. A aceitação favorável dos imigrantes haitianos em relação ao material educativo ressalta a importância de estratégias personalizadas, levando em conta suas particularidades individuais, étnicas, culturais e sociais. 

Em uma análise de autogerenciamento do Diabetes do tipo 2 (DM2), descobriu-se que os comportamentos de autocuidado são influenciados pela cultura do indivíduo, pelo apoio social que se recebe e pelas orientações que são repassadas para o autocuidado^47^. No caso dos imigrantes, é importante pensar a saúde na perspectiva da integralidade, dando suporte para que possam ter condições de autocuidado e acesso aos serviços de saúde, derrubando, principalmente, a barreira do idioma^7^ ^),(^ ^48^.

Em 2021, o Ministério da Saúde do Brasil lançou um Plano de Ações Estratégicas para o Enfrentamento das Doenças Crônicas Não Transmissíveis no Brasil 2021-2030, incluindo ações para controlar as doenças cardiovasculares, diabetes, câncer e doença respiratória crônica, pelo controle do fumo, inatividade física, alimentação inadequada e uso prejudicial de álcool^49^ ^)-(^ ^50^. Tendo em vista a tendência crescente da população haitiana com DCNT, especialmente com doenças cardiovasculares, que vêm ocorrendo nas últimas décadas^51^ ^)-(^ ^52^, tanto quanto as vulnerabilidades dos imigrantes haitianos em Manaus, há necessidade de inovações estratégicas para melhor atendê-la.

O déficit na capacidade para interpretar e utilizar informações em saúde pode repercutir na manutenção da DCNT já instalada ou o aparecimento delas. A implementação de ações de autocuidado provoca colaboração entre enfermeiro e indivíduo/família, de forma que eles desenvolvam capacidades e conhecimentos para se adaptarem e procederem à tomada de decisão informada com relação à sua doença crônica^53^ ^)-(^ ^54^.

Para pessoas que vivem em condições crônicas, manejar tais condições envolve interação com informações sobre a doença assim como o diagnóstico, tratamento, mudanças de hábitos e uma melhor interação com os profissionais de saúde e o sistema de saúde^55^ ^)-(^ ^57^.

Em relação ao perfil do público-alvo, identificou-se indivíduos com menor estrato socioeconômico, em sua maioria mulheres pretas. Uma pesquisa aponta que indivíduos com tais características usualmente têm maiores dificuldades para acessar serviços e receber informações de saúde; e mulheres pretas têm probabilidade 8% maior de exibirem baixa compreensão das informações, concluindo que o estrato socioeconômico baixo e a cor da pele são fatores determinantes para o acesso e a utilização dos serviços de saúde^57^. 

Observando o letramento em saúde do público-alvo, contando com a participação de especialistas em saúde e usuários, a elaboração de tecnologias educativas promovem a coprodução de saúde e pode levar a uma melhoria na qualidade, segurança e justiça no cuidado, além de favorecer o aprendizado coletivo, entre outros benefícios^58^. 

Este estudo está sujeito a algumas limitações: 1) A amostra do grupo de imigrantes haitianos pode não ser tão representativa para a população de haitianos com Letramento em Saúde limitado e problemas com a leitura; e 2) Foram abordadas apenas quatro DCNT no intuito de evitar que o conteúdo se tornasse tão extenso.

Dessa forma, sugere-se que estudos futuros avaliem a eficácia de utilização desta tecnologia na rotina da população haitiana, direcionada às quatro DNCT que foram abordadas no Guia, concluindo as etapas do método com a fase de Aplicação da tecnologia. 

Sobre a relevância do estudo para a enfermagem, o Guia construído e validado, disponível em português e em crioulo, contribui na melhoria da comunicação, na derrubada de estereótipos e preconceitos relativos a essa população, no oferecimento de um atendimento mais humanizado, alinhando-se aos princípios de universalidade, integralidade e equidade do SUS. Ressalta-se que a presença de imigrantes de várias nacionalidades e idiomas no país requer do enfermeiro uma capacitação que torne a comunicação mais eficiente, assegurando que o imigrante seja compreendido e que as informações referentes à sua saúde sejam entregues de forma clara. Uma vez que isso não seja possível, principalmente em decorrência das barreiras linguísticas e culturais, outros dispositivos adaptados às necessidades dos imigrantes, devem ser disponibilizados e acessados por eles. 

Além disso, este estudo contribui de forma significativa para o desenvolvimento da ciência da enfermagem, ao produzir e validar um Guia sobre DCNT que reúne informações atualizadas, variadas e confiáveis, destinado a imigrantes haitianos. Seu uso possibilitará reduzir práticas inadequadas de autocuidado e prevenção. 

## Conclusão

O Guia foi elaborado a partir de diretrizes nacionais e internacionais sobre DCNT mais prevalentes na população haitiana, levantadas a partir de uma revisão integrativa de literatura, aprimorado a partir de sugestões dos participantes e de fontes científicas. Foi validado segundo conteúdo e semântica, cujo processo de avaliação incluiu 17 especialistas da área da saúde e 30 imigrantes haitianos residentes em Manaus, Amazonas.

Na validação do conteúdo, obteve-se IC geral de 91% com os três atributos (objetivos, estrutura/apresentação e relevância) avaliados como satisfatórios. Na validação da semântica, a pontuação geral alcançou 25,8 (99,2%) de um total de 26 pontos, referendando o Guia como satisfatoriamente adequado no que tange ao conteúdo, linguagem, ilustrações gráficas, motivação e adequação cultural do Guia.

A tecnologia, na língua crioula, avaliada como apropriada, poderá mediar ações educativas de prevenção e autocuidado de Doenças Crônicas Não Transmissíveis para imigrantes haitianos. Na versão final do guia, considerou-se a adequação cultural, buscando evitar vieses associados à compreensão e conceituação de problemas de saúde, normalmente vivenciados pelo público-alvo, ciente de que as adaptações culturais e linguísticas são barreiras potenciais para o acesso aos sistemas de saúde e à qualidade da atenção à saúde, e impactam os problemas relacionados à ocorrência das DCNT e suas complicações.

Ademais, esta pesquisa colabora com a transferência de conhecimento e disseminação de informações com população em situação de vulnerabilidade.

## Data Availability

Todos os dados gerados ou analisados durante este estudo estão incluídos neste artigo publicado.

# Translation: Tecnología educativa sobre enfermedades crónicas no transmisibles para inmigrantes haitianos en lengua criolla: estudio de validación*

**DOI:** 10.1590/1518-8345.8103.4920

**Authors:** Olbichoo Lexius, Maria de Nazaré de Souza Ribeiro, Cleisiane Xavier Diniz, Elizabeth Teixeira, Fátima Helena do Espírito Santo, Aldalice Aguiar de Souza

**Affiliations:** 1 Universidade do Estado do Amazonas, Escola Superior em Ciências da Saúde, Manaus, AM, Brasil.; 2 Universidade Federal da Bahia, Instituto de Saúde Coletiva, Salvador, BA, Brasil.; 3 Becaria de la Fundação de Amparo à Pesquisa do Estado do Amazonas (FAPEAM), Brasil.; 4 Universidade Federal Fluminense, Escola de Enfermagem Aurora de Afonso Costa, Niterói, RJ, Brasil.

**Keywords:** Enfermedades no Transmisibles, Emigrantes e Inmigrantes, Estudio de Validación, Tecnología Educacional, Enfermería, Educación en Salud

## Abstract

**Objetivo:**

validar el contenido y la semántica de una tecnología educativa en lengua criolla, en formato de guía, para la prevención y el autocuidado de Enfermedades Crónicas o Transmisibles en inmigrantes haitianos.

**Método:**

se trata de un estudio de validación de una tecnología educativa en lo referente a los atributos de contenido y semántica. Participaron en la validación de contenido 17 especialistas del área de la salud (versión en portugués) y en la validación semántica 30 inmigrantes haitianos (versión en lengua criolla). En la validación del contenido, se utilizó un instrumento que evaluó los dominios objetivos, estructura/presentación y relevancia del contenido de la guía mediante una escala ordinal de cuatro puntos, y en el análisis se buscó el Índice de Concordancia; en la validación semántica se utilizó el instrumento Suitability Assessment of Materials, y en el análisis se buscó la puntuación SAM.

**Resultados:**

en el atributo contenido el Índice de Concordancia general fue del 91%; en el atributo semántica la puntuación SAM obtenida fue de 25,8 puntos.

**Conclusión:**

la tecnología educativa obtuvo validación de contenido y semántica, lo que indica que es apropiada para mediar acciones educativas de prevención y autocuidado de Enfermedades Crónicas No Transmisibles entre inmigrantes haitianos.

## Destacados

**(1)** La Guía demostró relevancia tecnológica, evaluada como adecuada.

**(2)** La tecnología validada es una herramienta científica adaptada al contexto de los inmigrantes.

**(3)** La tecnología, en lengua criolla, podrá mediar acciones educativas para haitianos.

## Introducción

Las diferencias culturales y lingüísticas, las dificultades para lidiar con las normas de un sistema de salud desconocido, con diferentes reglas y rutinas, asociadas a las vulnerabilidades sociales derivadas del estatus indeterminado de ciudadanía, son factores importantes que dificultan la integración y el acceso a los servicios de salud por parte de los inmigrantes en Brasil, principalmente de aquellos que se comunican en una lengua bastante distinta del portugués, como ocurre con los haitianos, que tienen el criollo como lengua materna y el francés como lengua adicional.

En Brasil, los inmigrantes tienen derecho al acceso a los servicios de salud; sin embargo, la diversidad cultural y lingüística compromete la calidad de la atención ofrecida por los servicios de salud, como en cualquier otro lugar del mundo^1^ ^)-(^ ^3^. El idioma es una de las mayores limitaciones de los profesionales de la salud en la atención al inmigrante y una importante barrera de acceso de estos individuos al sistema de salud, haciendo inviable la comunicación para ambos^4^ ^)-(^ ^6^.

Las interacciones entre los procesos migratorios y la salud están marcadas por una complejidad y por múltiples factores, que hacen a los inmigrantes particularmente vulnerables a las dificultades enfrentadas. Es fundamental comprender y reconocer sus particularidades, además de promover mejoras en su calidad de vida, garantizar la igualdad en el acceso a la salud y asegurar la dignidad en la atención. Esto incluye acciones para combatir desigualdades, discriminaciones y estigmas, así como el desarrollo y la mejora de las políticas públicas. También es esencial ofrecer servicios que atiendan a las necesidades actuales de los inmigrantes^7^ ^)-(^ ^10^.

Desde el año 2010, cuando uno de los terremotos más devastadores azotó Haití, la capital del estado de Amazonas pasó a recibir un gran flujo de inmigrantes haitianos. Estos individuos ingresaron a Brasil por las fronteras del norte y, posteriormente, migraron a otras partes del país en busca de oportunidades de trabajo e ingresos^9^ ^),(^ ^11^. Entre ellos se encuentran aquellos que no poseen visa permanente, con dificultades de comunicación (hablan el idioma criollo y pocos hablan francés), y que conforman un grupo social con diversos desafíos significativos para el Sistema Único de Saúde (SUS)^9^ ^),(^ ^12^.

Los inmigrantes, en general, son más vulnerables social y económicamente por someterse con mayor facilidad a trabajos insalubres, salarios bajos y viviendas inhóspitas. Tales factores propician un mayor riesgo de desnutrición, enfermedades laborales y enfermedades infecciosas como tuberculosis, hepatitis, VIH, Enfermedades Crónicas No Transmisibles (ECNT), además de trastornos psicosociales derivados de los cambios sociales y del alejamiento de la familia^8^ ^),(^ ^13^ ^)-(^ ^14^.

En relación con las ECNT, se destaca que son un conjunto de enfermedades con causas y factores de riesgo diversificados y modificables, con un período prolongado de latencia y de evolución, a menudo resultando en discapacidades funcionales. La evidencia señala que las muertes por ECNT se imputan a las enfermedades del sistema circulatorio (ESC), a las enfermedades crónicas del sistema respiratorio, al cáncer y a la diabetes^15^ ^)-(^ ^16^. En este ámbito, caben iniciativas de educación en salud a favor del autocuidado.

La educación en salud, como importante estrategia de difusión de información y orientaciones, ayuda al individuo, la familia y la comunidad a mejorar su salud, enriqueciendo sus conocimientos y afectando su actitud en relación con el bienestar y el autocuidado^17^ ^)-(^ ^19^. El autocuidado es una función humana reguladora que las personas desempeñan por sí mismas o que es realizada por ellas a partir de orientación, para preservar la vida, la salud, el desarrollo y el bienestar^20^ ^)-(^ ^21^. Promover y mantener el autocuidado en personas afectadas por ECNT es un papel fundamental de la actuación de los profesionales de la salud, principalmente enfermeros, por su interacción constante con el individuo. Para mediar la actuación de los profesionales, se puede hacer uso de tecnologías educativas en salud.

Las tecnologías educativas en salud podrán favorecer la autonomía, fortalecer la construcción del conocimiento y, así, configurarse como factibles para la promoción de la salud, la reducción de riesgos y la prevención de vulnerabilidades^22^ ^)-(^ ^24^. También pueden impulsar la educación en salud y proporcionar información esencial para disminuir dudas, a fin de cambiar actitudes de riesgo al ampliar el nivel de conocimiento de los sujetos involucrados^17^ ^),(^ ^25^ ^)-(^ ^27^.

Considerando tales perspectivas, se realizó una revisión de la literatura de tipo integrativa que buscó respuesta a la siguiente pregunta: ¿cuáles son las enfermedades crónicas no transmisibles más prevalentes en la población haitiana? Se seleccionaron 25 referencias que atendieron la pregunta de investigación, provenientes de 22 revistas. Se identificaron cuatro ECNT (las más prevalentes): hipertensión, diabetes, cáncer de mama y cáncer de cuello uterino. Con base en los resultados, se elaboró una tecnología educativa titulada “Guía de prevención y autocuidado de Enfermedades Crónicas No Transmisibles para inmigrantes haitianos, residentes en Manaus, Amazonas, Brasil”. La construcción se ancló en aspectos como claridad y lenguaje accesible, con el objetivo de hacer la guía atractiva y comprensible^28^.

Para la elaboración del contenido de la Guía, se optó por utilizar manuales y directrices oficiales nacionales e internacionales sobre ECNT de la Sociedade Brasileira de Diabetes, del Instituto Nacional de Câncer (INCA), de la Associação Brasileira para o Estudo da Obesidade e da Síndrome Metabólica (ABESO), de la Sociedade Brasileira de Cardiologia (SBC), del Ministério da Saúde, de la Organización Mundial de la Salud (OMS), de la Organización Panamericana de la Salud (OPS), de la Sociedade Brasileira de Mastologia (SBM), del U.S. Department of Health and Human Services, de los Centers for Disease Control and Prevention (CDC), de la American Diabetes Association (ADA), entre otros.

Una tecnología educativa debe ser adecuadamente elaborada, precisa, relevante, bien comprendida y validada antes de su utilización^29^ ^)-(^ ^31^. Una de las fases fundamentales es la validación, realizada mediante opiniones de especialistas en la temática (validación de contenido) y la comprensión del texto por el público objetivo (validación semántica)^29^ ^),(^ ^31^ ^)-(^ ^33^.

Considerando lo expuesto, se formuló la siguiente pregunta de investigación: ¿la tecnología elaborada, en lo referente al contenido (versión en portugués) y a la semántica (versión en lengua criolla), es adecuada para ser utilizada? En este sentido, el estudio tuvo como objetivo validar el contenido y la semántica de una tecnología educativa sobre prevención y autocuidado de Enfermedades Crónicas No Transmisibles para inmigrantes haitianos.

## Método

### Tipo de estudio

Se trata de un estudio de validación de una tecnología educativa según los atributos de contenido (versión en portugués) y semántica (versión en lengua criolla)^33^. Tras la validación de la versión en portugués, una de las investigadoras (haitiana) realizó la traducción a la lengua criolla y cinco hablantes nativos de la lengua realizaron la revisión lingüística. Después de ambas validaciones, se procedió a la elaboración de las dos versiones finales de la Guía.

### Escenarios del estudio

La validación de contenido se realizó en un entorno virtual. La validación semántica se desarrolló de forma presencial en la sede de la Associação Fanm Nwa, una asociación de mujeres negras, inmigrantes de Haití, que participan en la asociación con sus familiares y otros inmigrantes haitianos. La elección de la Associação Fanm Nwa se debió a su representatividad, por ser la única asociación oficial de inmigrantes haitianos en el estado de Amazonas, y por contar con un registro de inmigrantes. La asociación se ubica en la ciudad de Manaus-AM. 

### Período

El proceso de validación de contenido (versión en portugués) se llevó a cabo de enero a febrero de 2023; la revisión y traducción de la Guía a la lengua criolla ocurrió en marzo del mismo año, seguida de la validación semántica (versión en lengua criolla) de abril a mayo; la difusión pública de las dos versiones finales de la Guía fue en julio de 2023. 

### Criterios de selección

Para la validación de contenido se seleccionaron profesionales del área de la salud con énfasis en salud de la familia, salud colectiva y salud pública (especialistas). La búsqueda se realizó mediante la Plataforma Lattes. Se seleccionaron aquellos que cumplieron al menos dos de los criterios listados: tener como mínimo tres años de experiencia en la atención primaria; tener producción científica en el área; haber participado en eventos en los últimos cinco años; ser especialista, máster o doctor en el área de salud de la familia, salud pública, salud colectiva o áreas afines (cardiología, nefrología, endocrinología).

Para la validación semántica se seleccionaron inmigrantes haitianos (público objetivo) residentes en la ciudad de Manaus-AM y registrados en la Associação Fanm Nwa. Se seleccionaron aquellos con al menos 18 años y que supieran leer en lengua criolla. Como criterios de exclusión para ambos grupos de participantes, se considerarían no elegibles aquellos que: permanecieran sin responder hasta el día 15 después de aceptar la invitación; comunicaran a la coordinación del proyecto manifestación de sufrimiento psíquico durante el proceso; presentaran dificultades de comprensión inherentes a los procesos de validación.

### Definición de la muestra

Para definir el tamaño de la muestra para la validación de contenido (especialistas) y la validación semántica (público objetivo), se siguió lo recomendado por Pasquali, es decir, entre 6 y 20 participantes^34^.

### Variables del estudio

En relación con los especialistas, se consideraron las siguientes variables: edad, género, formación académica, cualificación profesional y experiencia en el área. Para el público objetivo: edad, género, ocupación, nivel de instrucción.

### Recolección de datos e instrumentos utilizados

Los especialistas fueron invitados mediante contacto electrónico (correo electrónico obtenido en la sección de contacto de la Plataforma Lattes) mediante una carta de invitación que contenía los objetivos del estudio, la justificación y la importancia de su contribución. A quienes respondieron positivamente, se les envió el enlace de la herramienta Google Forms^®^ que contenía: el Consentimiento Informado (CI), una versión de la guía en portugués, el instrumento e información sobre el llenado de los documentos y el envío de sugerencias. Tras el envío del enlace, se otorgó un plazo de quince días para la devolución. Los especialistas que no respondieron en el tiempo estipulado fueron excluidos.

El público objetivo fue invitado por el equipo de investigación durante reuniones presenciales en la sede de la Associação Fanm Nwa. Se les abordó de forma oral, momento en el que recibieron explicaciones sobre los objetivos del estudio, la justificación y la importancia de su contribución. Tras manifestar su aceptación, recibieron la información necesaria para la participación y, en formato impreso, el CI y una versión de la guía en lengua criolla. El llenado del instrumento y la indicación de sugerencias se realizaron después de la presentación proyectada de la guía y su lectura individual, llevados a cabo en tres encuentros de fin de semana, específicamente con este fin.

El instrumento utilizado para la validación de contenido (adaptado de investigadores brasileños)^35^ evaluó los dominios objetivos, estructura/presentación y relevancia del contenido de la guía mediante una escala ordinal de cuatro puntos: totalmente adecuado, adecuado, parcialmente adecuado e inadecuado. El instrumento contaba con un espacio para la inclusión de sugerencias. La adaptación realizada fue en la redacción de los ítems de los dominios.

El instrumento para la evaluación semántica [Suitability Assessment of Materials (SAM) adaptado por Galdino^36^] evaluó la guía en cinco dominios (contenido; lenguaje; ilustraciones gráficas; adecuación cultural y motivación) con 13 ítems con tres puntuaciones: 0 (inadecuado), 1 (parcialmente inadecuado) y 2 (adecuado). En el instrumento había espacio para la inclusión de sugerencias.

### Tratamiento y análisis de los datos

Los datos obtenidos con ambos instrumentos se registraron en una hoja de cálculo elaborada en Excel con doble verificación para controlar posibles errores de carga de la base de datos. Posteriormente, se trasladaron al *software* estadístico IBM^®^ *Statistical Package for the Social Sciences*, versión 24.0. En el análisis de los datos obtenidos con el instrumento de validación de contenido, se buscó el Índice de Concordancia (IC) en cuanto a la representatividad respecto del contenido por dominio (se sumaron los ítems evaluados como “TA” y “A” y se dividió por el número total de respuestas del dominio abordado) y el valor global (obtenido a partir de la media aritmética del IC por dominio). Se consideró como parámetro mínimo de validación de contenido alcanzar el 70% de IC^33^. La media y la desviación estándar se calcularon para variables continuas, y la frecuencia absoluta y relativa para las variables. En el análisis de los datos obtenidos con el instrumento SAM, se buscó la Puntuación SAM^34^. Se consideró una puntuación igual o superior a 10 puntos del total de 26 puntos^33^. Las sugerencias obtenidas en ambas etapas de validación se organizaron en un cuadro a partir de la mención de las siguientes acciones: incluir, modificar, reforzar, revisar. 

### Consideraciones éticas

Se cumplieron todos los requisitos éticos establecidos en la Resolución nº 466/2012, Resolución nº 510/2016 y la Carta Circular nº 1, de 3 de marzo de 2021, que trata de orientaciones para procedimientos en investigaciones con cualquier etapa en entorno virtual del Conselho Nacional de Saúde. El estudio fue evaluado y aprobado con el Dictamen 5.859.857 por el Comité de Ética en Investigación de la Escola Superior de Ciências da Saúde de la Universidade do Estado do Amazonas, mediante la Plataforma Brasil (CAAE 66058522.0.0000.5016).

## Resultados

En la validación de contenido participaron 17 profesionales, siendo 16 enfermeras(os) y un médico; todos con especialización en atención primaria, salud pública, salud colectiva y de la familia. En cuanto a la formación académica, cuatro especialistas, seis magísteres y siete doctores; el tiempo de experiencia práctica en las respectivas áreas fue de cuatro a 39 años.

En el dominio objetivo (que evalúa propósitos, metas o fines que se desea alcanzar con la tecnología), el IC fue del 93%. En el dominio estructura y presentación (que evalúa la organización general, estructura, estrategia de presentación, coherencia y formato) el IC alcanzó el 87%. Se constató que este dominio presentó el menor IC, ya que 15 respuestas obtuvieron PA (parcialmente adecuado). El dominio relevancia (que evalúa el grado de significación de la tecnología) alcanzó un IC del 94%. En el cálculo general se sumaron los dominios I, II y III y se dividieron por el número de dominios, obteniéndose un IC general del 91%. Se constató que todos los dominios alcanzaron un IC satisfactorio, lo que indica adecuación del contenido (Tabla 1).

**Tabla 1:** Distribución de las puntuaciones e IC* de las respuestas obtenidas de los especialistas del área de la salud en cada dominio. Manaus, AM, Brasil, 2023

1-Objetivos - Se refieren a propósitos, metas o fines que se desea alcanzar						
Ítems del Dominio 1	TA^†^	A^‡^	PA^§^	I^||^	TA^†^+A^‡^	IC*
La información/los contenidos son coherentes con las necesidades cotidianas del público objetivo de la tecnología.	12	05	-	-	17	100%
La información/los contenidos son importantes para la calidad de vida y/o el trabajo del público objetivo de la tecnología.	15	02	-	-	17	100%
Invita y/o incentiva cambios de comportamiento y actitud.	10	05	02	-	15	88%
Puede circular en el ámbito científico del área.	09	07	01	-	16	97%
Responde a los objetivos de instituciones que trabajan con el público objetivo de la tecnología.	09	05	03	-	14	82%
Dominio 1 - Total do IC* = 93%						
**2-Estructura/Presentación - Incluye organización general, estructura, estrategia de presentación, coherencia y formato.**						
**Ítems del Dominio 2**	**TA** ^†^	**A** ^‡^	**PA** ^§^	**I** ^||^	**TA** ^†^+A^‡^	IC*
La tecnología es apropiada para el público objetivo.	11	05	01	-	16	94%
Los mensajes están presentados de manera clara y objetiva.	09	07	01	-	16	94%
La información presentada es científicamente correcta.	05	10	02	-	15	88%
El material es apropiado al nivel sociocultural del público objetivo.	08	06	03	-	14	82%
Existe una secuencia lógica del contenido propuesto.	11	05	01	-	16	94%
La información está bien estructurada en cuanto a concordancia y ortografía.	07	04	06	-	11	64%
El estilo de redacción corresponde al nivel de conocimiento del público objetivo.	07	09	01	-	16	94%
Dominio 2 - Total do IC* = 87%						
**3-Relevancia - grado de significación de la tecnología**						
**Ítems del Dominio 3**	**TA** ^†^	**A** ^‡^	**PA** ^§^	**I** ^||^	**TA** ^†^+A^‡^	IC*
Los temas retratan aspectos clave que deben reforzarse.	13	03	01	-	16	94%
La tecnología permite la generalización y la transferencia del aprendizaje a diferentes contextos.	13	03	01	-	16	94%
La tecnología propone la construcción de conocimientos.	13	02	02	-	15	88%
La tecnología aborda los asuntos necesarios para el conocimiento del público objetivo.	12	04	01	-	16	94%
La tecnología es adecuada para ser utilizada por cualquier profesional con el público objetivo.	08	09	-	-	17	100%
Dominio 3 - Total do IC* = 94%						
$IC*\;\text{GENERAL}:\frac{93\%\left(\text{Dominio}\;1\right)+87\%\left(\text{Dominio}\;2\right)+94\%\left(\text{Dominio}\;3\right)}{3}=91\%$						

*IC = Índice de Concordancia; ^†^TA = Totalmente Adecuado; ^‡^A = Adecuado; ^§^PA = Parcialmente Adecuado; ^||^I = Inadecuado

Las sugerencias obtenidas en la validación de contenido se organizaron según acciones^37^ a ejecutarse: incluir, modificar, reforzar y revisar (Figura 1). Todas las sugerencias fueron aceptadas y consideradas para la elaboración de la versión final (portugués).

**Figura 1:** Distribución de las sugerencias de los especialistas del área de la salud según acciones a realizar en el texto (versión en portugués). Manaus, AM, Brasil, 2023

Atributo Contenido	Incluir:
	Información del Caderno de Atenção Básica nº 13, página 94, con relación a la participación de la mujer en la detección precoz del cáncer de mama (ES*- 04)
	El autoexamen de rutina publicado por el INCA^†^ (Breast awareness) (ES*- 05)
	Añadir algo gráfico/artístico que comunique identificación cultural para el haitiano (colores de la bandera, símbolo del país u otra forma de aproximación visual cultural) (ES*- 07)
	Poner a disposición en la guía las direcciones de las Unidades Básicas de Saúde, según la zona de Manaus, para seguimiento de salud (ES*- 15).
	En la página 20 colocar una imagen que muestre cómo medir la presión, así como se hizo en la aplicación de insulina (ES*-13)

	Modificar:
	En el tema DM2^‡^, sugiero que los factores de riesgo se mencionen antes de los signos y síntomas (ES*-08)

	Reforzar:
	Ser más objetiva y didáctica en las orientaciones, pues hay mucha escritura. Cuanto más visual sea la información, mejor será el entendimiento (ES*-16)
	Abordar más la Atención Primaria de Salud, puerta de entrada del Sistema Único de Salud, donde el usuario podrá aclarar dudas sobre la tecnología de salud y su autocuidado (ES*-12)

	Revisar:
	Términos como Hipertensión Arterial Sistémica y no solo Hipertensión Arterial, y revisión del portugués (ES*- 12)

*ES = Especialista del Área de la Salud; ^†^INCA = Instituto Nacional do Câncer; ^‡^DM2 = Diabetes Mellitus Tipo 2

En la validación semántica participaron 30 inmigrantes haitianos, 18 mujeres y 12 hombres, en el rango de edad entre 25 y 71 años, siendo 13 personas en el rango de 51 a 71 años. Entre ellos, 10 tenían el equivalente a la educación secundaria incompleta; solo uno, educación secundaria completa; cinco personas tenían educación primaria completa, 12 tenían educación primaria incompleta y dos, educación superior. En lo que se refiere a las actividades laborales, 14 eran vendedores ambulantes, seis realizaban servicios diversos (limpieza, embellecimiento y costura), dos profesoras, dos empresarias, dos albañiles y cuatro no realizaban ninguna actividad laboral remunerada. En relación con el tiempo en Brasil, 15 informaron residir en el país desde hacía 3 a 5 años, tiempo considerado corto; 13 residían entre 6 y 10 años; y solo dos de ellos residían en el país desde hacía más tiempo, entre 11 y 13 años.

En la validación semántica, de quienes evaluaron, 25 participantes asignaron 26 puntos (puntuación máxima) y cinco de ellos asignaron 25 puntos. La puntuación general fue 775 y el promedio de la puntuación individual alcanzó 25,8 puntos (99,2%). Se constató, a partir de los resultados, la adecuación semántica de la guía (Tabla 2).

**Tabla 2:** Distribución de las puntuaciones atribuidas por los inmigrantes haitianos de acuerdo con las respuestas obtenidas en cada ítem, según contenido, lenguaje, ilustraciones gráficas, motivación y adecuación cultural. Manaus, AM, Brasil, 2023

Ítems del dominio	00	01	02	Puntos obtenidos
	I*	PA^†^	A^‡^	
**Dominio 01 - *Tèks* / Contenido**				
1.1 Objektif la klè, li pèmèt ou konprann rapid sa ki nan gid la (El objetivo es evidente, lo que facilita la comprensión inmediata del material.).	-	-	60	60
1.2 Tèks yo abòde enfòmasyon ki gen rapò ak kòman pou evite maladi kwonik non transmisib, ak kòman pou w pran swen tèt ou si w ta genyen yo (El contenido aborda información relacionada que ayuda a prevenir y a realizar el autocuidado de las Enfermedades Crónicas No Transmisibles).	-	-	60	60
1.3 Gid la rive konplete objektif yo, pou lektè a ka konprann sa ki ekri yo nan yon tan ki rezonab epitou san konfizyon (La propuesta del material se limita a los objetivos, para que el lector pueda comprenderla en el tiempo permitido y sin confusión).	-	-	60	60
Total 180 puntos (100%)				
**Dominio 02 - *Langaj* / Lenguaje**				
2.1 Kantite fraz ki nan tèks yo bon pou pèmèt imigran yo konprann (El nivel de lectura es adecuado para la comprensión del inmigrante haitiano).	-	-	60	60
2.2 Jan tèks la ekri a pèmèt li fasil pou yo konprann (El estilo de escritura facilita la comprensión de los textos).	-	-	60	60
2.3 Vokabilè ki itilize yo se mo ke yo konnen e nou mete siyifikasyon (El vocabulario utiliza palabras comunes).	-	-	60	60
Total 180 puntos (100%)				
**Dominio 03 - *Reprezantasyon Grafik* / Ilustraciones Gráficas**				
3.1 Tit la kapte atansyon epi montre objektif gid la (El título atrae la atención y refleja el propósito del material).	-	-	60	60
3.2 Imaj yo montre mesaj yo klè pou lektè a ka konprann pwen prensipal yo pou kont li, san li pa distrè (Las ilustraciones presentan mensajes visuales fundamentales para que el lector pueda comprender los puntos principales por sí solo, sin distracciones).	-	04	52	56
Total 116 puntos (96,7%)				
**Dominio 04 - *Motivasyon* / Motivación**				
4.1 Tèks yo kominike ak lektè a. Li ap pèmèt yo rezoud pwoblèm yo, fè chwa ak / oswa montre kapasite yo (Ocurre interacción del texto con el lector, llevándolos a resolver problemas, tomar decisiones y/o demostrar habilidades).	-	01	58	59
4.2 Modèl konpòtman ou vle yo byen reprezante oswa byen klè (Los patrones de comportamiento deseados están modelados o bien demostrados).	-	-	60	60
4.3 Gen enterè pou rann pwòp tèt yo itil, sa vle di, yo motive moun yo pou yo aprann paske yo kwè ke objektif ak konpòtman yo posib (Existe motivación hacia la autoeficacia, es decir, las personas están motivadas a aprender por creer que las tareas y los comportamientos son factibles).	-	-	60	60
Total 179 puntos (99,4%)				
**Dominio 05 - *Akò Kiltirèl* / Adecuación Cultural**				
5.1 Nan lojik kiltirèl, nan lang ak ekperyans imigran yo gid la byen konstwi (El material es culturalmente adecuado a la lógica, el lenguaje y la experiencia del público objetivo).	-	-	60	60
5.2 Li prezante imaj ak mo ki annakò ak kilti yo (Presenta imágenes y ejemplos culturalmente adecuados).	-	-	60	60
Total 120 puntos (100%)				
$\text{Puntuación General}\;\frac{775\;\text{puntos}}{30}=25,8\left(99,2\%\right)$				

*I = Inadecuado; ^†^PA = Parcialmente Adecuado; ^‡^A = Adecuado

Las sugerencias obtenidas en la validación semántica también se organizaron según acciones^37^ a ejecutarse: modificar y reforzar (Figura 2). Todas las sugerencias fueron aceptadas y consideradas para la elaboración de la versión final (lengua criolla).

**Figura 2:** Distribución de las sugerencias de los inmigrantes según acciones a realizar en el texto (versión en lengua criolla). Manaus, AM, Brasil, 2023

Atributo Semántica	Modificar:
	Mete mo ‘’natirèl’’ oswa ‘’Bio’’ nan plas ‘’Entegral’’(Colocar la palabra “natural” o “Bio” en lugar de “Integral”) (IH*- 02)

	Reforzar:
	Mwen sijere ou konstwi pwochèn gid, nenpòt zouti an ak yon nitrisyonis pou w ka mete ki fri espesifik moun ki fè maladi sik oswa tansyon wo pa ka manje paske mwen tande gen fri ki monte sik ak tansyon. (Cuando desarrolle la próxima Guía o cualquier herramienta, hágalo junto con un nutricionista, a fin de incluir las frutas específicas que las personas con diabetes o hipertensión no pueden comer, porque escuché que hay frutas que aumentan la glucemia y la presión arterial) (IH*- 02)

*IH = Inmigrante Haitiano

La versión final, tras ambas validaciones, se estructuró con los siguientes elementos: portada, ficha catalográfica, equipo de elaboración, presentación de la Guía, descripción de las cuatro ECNT (Hipertensión Arterial, Diabetes, Cáncer de Mama y Cáncer de Cuello Uterino/Cáncer Cervical), consideraciones finales y referencias. Se enfatizó la información referente a: prevención de las ECNT, signos y síntomas, complicaciones, tratamiento, recomendaciones para el control de la enfermedad, acceso a la medicación y un enlace para acceder a las direcciones de las unidades de salud más cercanas. En espacios específicos de la Guía, se destacó que la Constitución Federal Brasileña y el SUS garantizan el acceso universal a la salud para los inmigrantes. Se utilizó un lenguaje accesible para facilitar la comprensión por parte del público objetivo; ilustraciones gráficas representativas de la cultura haitiana; y organización textual e iconográfica para motivar el uso de la Guía, de forma creativa y proactiva, a fin de que hubiera involucramiento del sujeto^1^ ^)-(^ ^2^ ^),(^ ^25^.

Para complementar los textos, se utilizaron figuras elaboradas de forma manual en Figma^®^, por una diseñadora gráfica contratada, además de otras imágenes obtenidas del acervo de Canva^®^, garantizando que el contenido fuera adaptado y visualmente alineado con el propósito educativo y cultural identitario (Figura 3). La presencia de representatividad étnico-racial en las tecnologías de salud, especialmente de la comunidad negra, puede contribuir a una mejora del autocuidado y aumentar la adherencia a los tratamientos^38^ ^)-(^ ^39^.

**Figura 3:** Imagen ilustrativa de la Guía en lengua criolla titulada “Gid prevansyon ak swen pèsonèl Maladi Kwonik Non Transmisib pou imigran ayisyen nan Amazonas, peyi Brezil”

A fin de dar visibilidad y provocar una mayor identificación, en la portada se colocaron las banderas de Brasil y de Haití; los colores azul y rojo, presentes en la bandera de Haití, se mantuvieron en toda la apertura de cada tema y en las páginas siguientes (Figura 3).

La última versión de la Guía se finalizó con 73 páginas, debido al tamaño de la fuente (14) y a la inserción de otras imágenes sugeridas por los evaluadores. Las versiones finales en portugués y en lengua criolla, en formato PDF, están disponibles en: http://educapes.capes.gov.br/handle/capes/735019 y http://educapes.capes.gov.br/handle/capes/734982.

## Discusión

Es incontestable que los inmigrantes experimentan procesos de enfermedad con la aparición de las ECNT, como reflejo de los modos de vida y trabajo en contextos de vulnerabilidad social en los países receptores, que, de forma constante, comprenden situaciones de riesgo efectivas para la salud, características de su inserción como inmigrantes en la sociedad^7^.

En 2019, el International Council of Nurses (ICN) reiteró el papel de los enfermeros en el programa “Salud para Todos”, enfatizando su importancia en la determinación de las necesidades de salud de sus pacientes y en la enseñanza de maneras y prácticas saludables para garantizar que se encuentren en óptimas condiciones de salud. Así, los enfermeros y otros profesionales de la salud se encuentran en una excelente posición para efectuar cambios positivos en la vida de los individuos y de sus familias mediante la educación en salud, mejorando sus conocimientos, actitudes y comportamientos^40^.

La tecnología aquí validada se constituye como una herramienta planificada y desarrollada con base en evidencias científicas, en el contexto cultural y social de los inmigrantes haitianos que residen en Brasil, más específicamente en el estado de Amazonas, y en las necesidades de salud de este público. En la aplicabilidad de la Guía, se tomaron en consideración el público objetivo, el escenario y el contexto^1^ ^),(^ ^3^ ^)-(^ ^4^ ^),(^ ^6^ ^),(^ ^9^. Se enfatiza que todas las sugerencias procedentes se incorporaron en la segunda versión de la Guía, como se indica en la literatura^32^ ^)-(^ ^33^ ^),(^ ^36^.

Los resultados generales de la validación de contenido de la Guía demostraron concordancia y relevancia de la tecnología, evaluándola como adecuada, al obtener una puntuación del 91%, superior al mínimo establecido (70%). Estudios similares sobre diabetes, hipertensión, cáncer de mama y cáncer cervical alcanzaron IC del 84%^41^, 84%^42^, 91,71^43^, 95%^44^, respectivamente.

En relación con la validación semántica, la Guía fue considerada de calidad superior, con una puntuación total del 99,2%, por encima del mínimo establecido (70%). Estudios que utilizaron el mismo instrumento para la validación semántica de tecnologías educativas en salud obtuvieron valores muy próximos^34^ ^),(^ ^44^ ^)-(^ ^46^. La aceptación favorable de los inmigrantes haitianos respecto del material educativo resalta la importancia de estrategias personalizadas, teniendo en cuenta sus particularidades individuales, étnicas, culturales y sociales.

En un análisis de automanejo de la Diabetes tipo 2 (DM2), se descubrió que los comportamientos de autocuidado están influidos por la cultura del individuo, por el apoyo social que recibe y por las orientaciones que se transmiten para el autocuidado^47^. En el caso de los inmigrantes, es importante pensar la salud en la perspectiva de la integralidad, brindando soporte para que puedan contar con condiciones de autocuidado y acceso a los servicios de salud, derribando, principalmente, la barrera del idioma^7^ ^),(^ ^48^.

En 2021, el Ministerio de Salud de Brasil lanzó un Plan de Acciones Estratégicas para el Enfrentamiento de las Enfermedades Crónicas No Transmisibles en Brasil 2021-2030, que incluye acciones para controlar las enfermedades cardiovasculares, la diabetes, el cáncer y la enfermedad respiratoria crónica, mediante el control del tabaquismo, la inactividad física, la alimentación inadecuada y el uso perjudicial de alcohol^49^ ^)-(^ ^50^. Teniendo en cuenta la tendencia creciente de la población haitiana con ECNT, especialmente con enfermedades cardiovasculares, que vienen ocurriendo en las últimas décadas^51^ ^)-(^ ^52^, así como las vulnerabilidades de los inmigrantes haitianos en Manaus, existe la necesidad de innovaciones estratégicas para atenderla mejor.

El déficit en la capacidad para interpretar y utilizar información en salud puede repercutir en el mantenimiento de una ECNT ya instalada o en su aparición. La implementación de acciones de autocuidado promueve la colaboración entre el enfermero y el individuo/familia, de modo que desarrollen capacidades y conocimientos para adaptarse y proceder a la toma de decisiones informadas con relación a su enfermedad crónica^53^ ^)-(^ ^54^.

Para las personas que viven con condiciones crónicas, el manejo de dichas condiciones implica interacción con información sobre la enfermedad, así como el diagnóstico, el tratamiento, cambios de hábitos y una mejor interacción con los profesionales de la salud y el sistema de salud^55^ ^)-(^ ^57^.

En relación con el perfil del público objetivo, se identificaron individuos de estrato socioeconómico más bajo, en su mayoría mujeres negras. Una investigación señala que individuos con tales características usualmente tienen mayores dificultades para acceder a servicios y recibir información de salud; y las mujeres negras tienen una probabilidad 8% mayor de presentar baja comprensión de la información, concluyendo que el estrato socioeconómico bajo y el color de piel son factores determinantes para el acceso y la utilización de los servicios de salud^57^.

Observando la competencia en salud del público objetivo, contando con la participación de especialistas en salud y usuarios, la elaboración de tecnologías educativas promueve la coproducción de salud y puede llevar a una mejora en la calidad, seguridad y justicia en la atención, además de favorecer el aprendizaje colectivo, entre otros beneficios^58^.

Este estudio está sujeto a algunas limitaciones: 1) La muestra del grupo de inmigrantes haitianos puede no ser tan representativa de la población haitiana con competencia en salud limitada y problemas de lectura; y 2) Se abordaron solo cuatro ECNT con el fin de evitar que el contenido se volviera demasiado extenso.

De esta forma, se sugiere que estudios futuros evalúen la eficacia del uso de esta tecnología en la rutina de la población haitiana, dirigida a las cuatro ECNT que se abordaron en la Guía, concluyendo las etapas del método con la fase de Aplicación de la tecnología.

Sobre la relevancia del estudio para la enfermería, la Guía construida y validada, disponible en portugués y en criollo, contribuye a mejorar la comunicación, a derribar estereotipos y prejuicios relativos a esta población, a ofrecer una atención más humanizada, alineándose con los principios de universalidad, integralidad y equidad del SUS. Se resalta que la presencia de inmigrantes de varias nacionalidades e idiomas en el país requiere del enfermero una capacitación que haga la comunicación más eficiente, asegurando que el inmigrante sea comprendido y que la información referente a su salud sea entregada de forma clara. Cuando esto no sea posible, principalmente debido a las barreras lingüísticas y culturales, otros dispositivos adaptados a las necesidades de los inmigrantes deben estar disponibles y ser accesibles para ellos.

Además, este estudio contribuye de forma significativa al desarrollo de la ciencia de la enfermería, al producir y validar una Guía sobre ECNT que reúne información actualizada, variada y confiable, destinada a inmigrantes haitianos. Su uso permitirá reducir prácticas inadecuadas de autocuidado y prevención.

## Conclusión

La Guía se elaboró a partir de directrices nacionales e internacionales sobre las ECNT más prevalentes en la población haitiana, identificadas mediante una revisión integrativa de la literatura, y se perfeccionó a partir de sugerencias de los participantes y de fuentes científicas. Fue validada según contenido y semántica, cuyo proceso de evaluación incluyó a 17 especialistas del área de la salud y a 30 inmigrantes haitianos residentes en Manaus, Amazonas.

En la validación del contenido, se obtuvo un IC general del 91%, con los tres atributos (objetivos, estructura/presentación y relevancia) evaluados como satisfactorios. En la validación de la semántica, la puntuación general alcanzó 25,8 (99,2%) de un total de 26 puntos, refrendando que la Guía es satisfactoriamente adecuada en lo que respecta al contenido, lenguaje, ilustraciones gráficas, motivación y adecuación cultural de la Guía.

La tecnología, en lengua criolla, evaluada como apropiada, podrá mediar acciones educativas de prevención y autocuidado de Enfermedades Crónicas No Transmisibles para inmigrantes haitianos. En la versión final de la guía, se consideró la adecuación cultural, procurando evitar sesgos asociados a la comprensión y conceptualización de problemas de salud, habitualmente vivenciados por el público objetivo, conscientes de que las adaptaciones culturales y lingüísticas son barreras potenciales para el acceso a los sistemas de salud y a la calidad de la atención en salud, e impactan los problemas relacionados con la ocurrencia de las ECNT y sus complicaciones.

Además, esta investigación contribuye a la transferencia de conocimiento y a la difusión de información con población en situación de vulnerabilidad.

## Data Availability

Todos los datos generados o analizados durante este estudio están incluidos en este artículo publicado.
